# Performance of Møller-Plesset second-order perturbation theory and density functional theory in predicting the interaction between stannylenes and aromatic molecules

**DOI:** 10.1007/s00894-015-2589-1

**Published:** 2015-02-13

**Authors:** Piotr Matczak, Sławomir Wojtulewski

**Affiliations:** 1 Department of Theoretical and Structural Chemistry, Faculty of Chemistry, University of Łódź, Pomorska 163/165, 90-236 Lodz, Poland; 2 Institute of Chemistry, University of Białystok, Hurtowa 1, 15-399 Bialystok, Poland

**Keywords:** Benchmarking, MP2, DFT, Benzene, Pyridine, Stannylene

## Abstract

The performances of Møller-Plesset second-order perturbation theory (MP2) and density functional theory (DFT) have been assessed for the purposes of investigating the interaction between stannylenes and aromatic molecules. The complexes between SnX_2_ (where X = H, F, Cl, Br, and I) and benzene or pyridine are considered. Structural and energetic properties of such complexes are calculated using six MP2-type and 14 DFT methods. The assessment of the above-mentioned methods is based on the comparison of the structures and interaction energies predicted by these methods with reference computational data. A very detailed analysis of the performances of the MP2-type and DFT methods is carried out for two complexes, namely SnH_2_-benzene and SnH_2_-pyridine. Of the MP2-type methods, the reference structure of SnH_2_-benzene is reproduced best by SOS-MP2, whereas SCS-MP2 is capable of mimicking the reference structure of SnH_2_-pyridine with the greatest accuracy. The latter method performs best in predicting the interaction energy between SnH_2_ and benzene or pyridine. Among the DFT methods, ωB97X provides the structures and interaction energies of the SnH_2_-benzene and SnH_2_-pyridine complexes with good accuracy. However, this density functional is not as effective in reproducing the reference data for the two complexes as the best performing MP2-type methods. Next, the DFT methods are evaluated using the full test set of SnX_2_-benzene and SnX_2_-pyridine complexes. It is found that the range-separated hybrid or dispersion-corrected density functionals should be used for describing the interaction in such complexes with reasonable accuracy.

**Electronic supplementary material:**

The online version of this article (doi:10.1007/s00894-015-2589-1) contains supplementary material, which is available to authorized users.

## Introduction

Quantum-chemical modeling of organometallic systems and inorganic systems containing metal atoms is a challenging task because of the large number of electrons and strong electron correlation effects occurring in metal atoms. In recent years the vast majority of computational studies of these systems were performed using density functional theory (DFT) [1, 2] and such a widespread adoption of DFT methods stemmed from their excellent ratio of computational cost to accuracy. DFT methods are able to include a large fraction of electron correlation and their computational cost scales favorably with the size of molecular systems (formally between O(*N* ^3^) and O(*N* ^4^), where *N* is proportional to system size). Unfortunately, these methods also have their weaknesses. DFT methods suffer from the spurious self-interaction of electrons, which results in too much electron delocalization and too low total energies [3, 4]. Another weakness is their inability to describe long-range electron correlations that are responsible for dispersive forces [1]. However, the constant development in DFT has led to some progress in overcoming, at least partially, these weaknesses. For instance, some reduction in the self-interaction can be provided by the inclusion of either the long-range correction or a substantial, global portion of exact exchange [3], whereas the lack of dispersion can be compensated by various empirical corrections [5]. Because DFT is in fact a family of methods (in other words, density functionals), and each of them presents an approximate approach to the unknown exact density functional, the approximations used in a given DFT method affect its accuracy in modeling a specific molecular system. Therefore, each density functional requires testing prior to its application and many studies evaluating the performance of density functionals for various organometallic systems and inorganic systems containing metal atoms have been published, e.g., [6–10] to mention only a few of the most recent.

Before the advent of the immense popularity of DFT methods, organometallic systems and inorganic systems containing metal atoms were usually investigated using Møller-Plesset second-order perturbation theory (MP2) [11], that is the simplest and most economical method among post-Hartree-Fock wave function-based theories (WFT). In contrast to DFT, the MP2 method is free from the spurious self-interaction of electrons and naturally takes dispersion into account. However, the MP2 dispersion may exhibit a deficiency when the interaction between fragments of a molecular system is calculated. The resulting interaction energy may be overestimated noticeably because the MP2 method makes use of the uncoupled Hartree-Fock dispersion energy that lacks the repulsive intramolecular correlation correction [12, 13]. Many attempts to remove such an overestimation were made in recent years [14–18] and one of the most successful solutions is, perhaps, the spin-component scaling (SCS) [14] that introduces separate scaling coefficients for MP2 correlation energy contributions coming from parallel- and antiparallel-spin pairs of electrons. These scaling coefficients can be either deduced from theory [19] or obtained from parametrization against some benchmark data [14, 20]. Therefore, SCS schemes can be tailored to modeling a specific kind of systems [21, 22]. Although the conventional MP2 method is the most economical post-Hartree-Fock method, its computational cost scales as *O*(*N* ^5^), hence it is much higher than that of DFT methods. The resolution of the identity (RI) [23, 24] is a widely used technique making the MP2 method more computationally affordable. It leads to computational savings amounting to 1–2 orders of magnitude compared to conventional MP2 calculations. The application of the conventional MP2 method to organometallic systems and inorganic systems containing metal atoms revealed severe inaccuracies in the description of 3*d* transition metal complexes [25]. In such cases the aforementioned modifications of the conventional MP2 method may provide an improvement in accuracy and the SCS-MP2 variant indeed performs better than MP2 for complexes with transition metals [26, 27].

The progress made in the last decade in the development of MP2 variants and density functionals has encouraged us to inspect how accurately such new methods are able to model systems composed of a stannylene and an aromatic molecule. Stannylenes are singlet species of divalent tin with a formal 5s^2^5p^2^ valence electron configuration [28]. In the ground state they possess an electron lone pair and a vacant p-orbital on the Sn atom. The vacant p-orbital is responsible for their high reactivity whereas the lone pair is rather inert due to its high s-character [29]. Many stannylenes can be easily obtained in laboratory conditions and some of them (e.g., dihalogenated stannylenes) are stable enough to be handled under ambient conditions [29]. From the practical point of view, stannylenes have found several important applications. For instance, they can act as active single-site catalysts for the living polymerization of lactide [30, 31]. Stannylenes have also been investigated theoretically, using mostly DFT methods [32–38] and rarely at the MP2 level of theory [33, 39]. Recently, the interaction between stannylenes and aromatic rings has attracted much interest [40], although such an interaction was detected experimentally some time before, e.g., [41]. In the theoretical studies devoted to this subject [42, 43] some density functionals such as B3LYP and PBE, also combined with a dispersion correction, were employed. However, it is not clear whether the B3LYP functional, which is without any doubt the most popular DFT method, provides a sufficiently accurate description of systems containing stannylenes and aromatic molecules and which DFT method would be in general most appropriate for investigating such systems. In this work, we would like to assess quantitatively the performance of selected MP2 variants and density functionals belonging to several DFT generations for predicting geometrical and energetic parameters of computational model systems that include stannylenes SnX_2_ (where X = H, F, Cl, Br, I) in their π-complexes with benzene and in their σ-complexes with pyridine. On the one hand, the singlet spin multiplicity of stannylenes simplifies these model systems but on the other hand providing the accurate theoretical description of interactions with aromatic molecules may be a demanding task [44–46].

## Computational details

In the first part of this work we focus on the performance of the conventional MP2 method and its five modifications, namely SCS-MP2 [14], SOS-MP2 [20], FE2-MP2 [47], SCS(MI)-MP2 [48], and S2-MP2 [19]. The results calculated using these six MP2-type methods were compared with the results obtained from CCSD [49] and CCSD(T) [49]. The CCSD method was recently used for providing reference geometries of non-covalently interacting molecular complexes [50, 51]. Reference interaction energies between stannylenes and aromatic molecules in the investigated π- and σ-complexes were calculated using CCSD(T) because this method is commonly considered to be the “golden standard” for obtaining reference results for systems with intermolecular interactions [52]. All the MP2-type methods and CCSD(T) employed the RI technique and the frozen core approximation to calculate the electron correlation energy. TURBOMOLE 6.3.1 [53] was used to carry out the calculations involving the MP2-type and CCSD(T) methods. The CCSD optimizations were performed with GAUSSIAN 09 C.01 [54] because analytic gradients were available for CCSD in this software. The frozen core approximation was also used in the CCSD calculations.

In the second part of the work a set of density functionals belonging to various DFT generations is tested. This set includes both representatives of older DFT generations, such as the generalized gradient approximation (GGA) and global hybrids (GH), and some modern density functionals such as those making use of the local kinetic energy density (that is, the meta-GGA generation) and range-separated hybrids (RSH). The generations of density functionals, together with their representatives considered in this work, are listed in Table 1.

**Table 1:** Generations of density functionals and their representatives considered in this work

Generation	Representative
GGA	BP86 [92, 93], BLYP [80, 92]
meta-GGA	TPSS [94]
GH GGA	B3LYP [95, 96], B98 [97]
GH meta-GGA	TPSSh [98]
RSH GGA	ωB97X [59]
RSH meta-GGA	M11 [99]

Four older density functionals (BLYP, BP86, B3LYP, and B98) were taken into account because they are still widely used in the investigations of organotin molecules [55–57], including stannylenes [32, 33, 36–38], and, what is more important, we wanted to verify whether the addition of a DFT-D dispersion correction improved their performance. We employed the D2 dispersion correction [58] that is classified as the so-called simple *C*_6_ correction [5]. This correction was parametrized only for selected density functionals. Grimme determined the D2 parameters appropriate for BP86, BLYP, B3LYP, and TPSS [58], whereas the D2-type correction for the ωB97X functional was proposed by Chai and Head-Gordon [59]. In this work the aforementioned functionals combined with the D2 correction will be denoted by the suffix “-D”. We decided to use the B97-D density functional [58] as the dispersion-corrected version of B98, although the presence of the dispersion correction is not the only difference between these two functionals. The DFT calculations were performed with GAUSSIAN 09 C.01 (for BP86, BLYP, B3LYP, B98, B97-D, ωB97X, and ωB97X-D), GAUSSIAN 09 D.01 [60] (for BP86-D, BLYP-D, B3LYP-D, and M11) and TURBOMOLE 6.3.1 (for TPSS, TPSS-D, and TPSSh).

Both the WFT and the DFT methods were combined with two types of triple-ζ valence basis sets, namely cc-pVTZ [61], that belongs to the family of basis sets proposed by Dunning and co-workers, and def2-TZVP [62], that in turn is a representative of the family developed by Ahlrichs and co-workers. The def2-TZVP basis set assigns small-core relativistic pseudopotentials (covering 28 core electrons) to the Sn and I atoms in the SnX_2_ stannylenes. The variant of def2-TVZP with double polarization functions designed for correlated calculations (that is, def2-TZVPP) was used in the WFT calculations. In the case of the cc-pVTZ basis set its extension developed by Peterson [63] and denoted by cc-pVTZ-PP was employed for Sn and I. In the cc-pVTZ-PP basis set the core electrons of Sn and I are described by the pseudopotentials that are very similar to those adopted in def2-TZVP. Additionally, cc-pVTZ and def2-TZVP in their diffuse-augmented versions, aug-cc-pVTZ [61] and def2-TZVPD [64], respectively, were also used.

The optimized geometries of the SnX_2_-benzene π-complexes (where X = H, F, Cl, Br, I) and of the SnX_2_-pyridine σ-complexes were confirmed to be true local minima with the aid of vibrational frequency analysis. The interaction energy between SnX_2_ and benzene/pyridine in the optimized complexes was calculated in the supermolecular fashion. The sum of the total energies of isolated SnX_2_ and benzene/pyridine in their geometries found in the SnX_2_-benzene/SnX_2_-pyridine complexes was subtracted from the total energy of these complexes. In order to remove the basis-set superposition error (BSSE) from the values of interaction energy, the counterpoise correction proposed by Boys and Bernardi [65] was employed.

The comparison of the SnX_2_-benzene or SnX_2_-pyridine structures optimized by different methods was made using the minimized root-mean-square value of the residual distances (RMSD) between the corresponding atoms belonging to these structures. The values of RMSD were computed with HYPERCHEM 8.0 [66].

## Results and discussion

### Assessment of MP2-type methods

We start with assessing the performance of six MP2-type methods in predicting the geometries of two stannylene-aromatic molecule complexes. The simplest possible stannylene, SnH_2_, was selected to form its complexes with benzene and with pyridine. As no experimental characterization of the geometries of these complexes exists, we used the geometries optimized with the CCSD method as references for the assessment of the MP2-type methods. The very high computational cost of the CCSD optimizations was the reason to focus here only on the two complexes. The assessment of the MP2-type methods was performed separately for each of four basis sets mentioned in the previous section and the same basis set was combined both with MP2-type methods and with CCSD in order to optimize the geometries of the complexes and then to compare them.

**Fig. 1:**
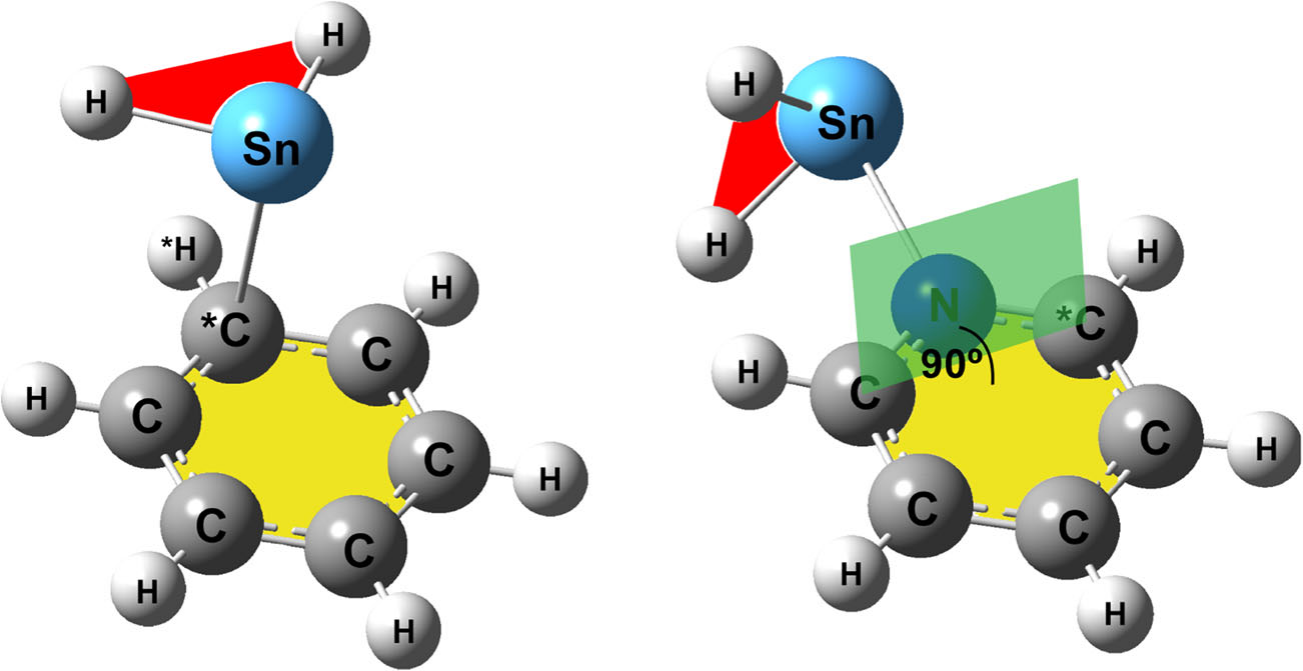
π-Complex of SnH_2_ with benzene (*left*) and σ-complex of SnH_2_ with pyridine (*right*)

Before we present the quantitative results of the assessment of the geometries optimized by six MP2-type methods, we give our attention to the SnH_2_-benzene and SnH_2_-pyridine complexes and describe roughly their structure itself. The optimization of the geometry of the system composed of SnH_2_ and benzene leads to a complex in which the Sn atom of SnH_2_ is positioned over one of the C atoms of benzene and two H atoms of the stannylene molecule are pointed to the outside of the benzene ring. The molecular plane of SnH_2_ is approximately parallel to the molecular plane of benzene (see Fig. 1, these two planes are colored red and yellow, respectively). In others words, the unfilled p-orbital of the Sn atom is pointed at the π-electron sextet and, therefore, the resulting complex can be termed the π-complex. As for the SnH_2_-pyridine complex, the stannylene molecule could interact with either the π-electron sextet or the lone electron pair of nitrogen. We investigated the second case and then the unfilled p-orbital of the Sn atom in SnH_2_ approached the lone electron pair on the N atom of pyridine. More specifically, the plane of the SnH_2_ molecule was arranged perpendicularly to the plane of pyridine (see Fig. 1, these two planes are marked red and yellow, respectively). The optimized geometries of both the π-complex of SnH_2_ with benzene and the σ-complex of SnH_2_ with pyridine exhibited the C_s_ point group symmetry. To conclude, SnH_2_-benzene and SnH_2_-pyridine will be considered here to be prototype systems for the π- and σ-complexations of stannylenes by aromatic molecules.

**Fig. 2:**
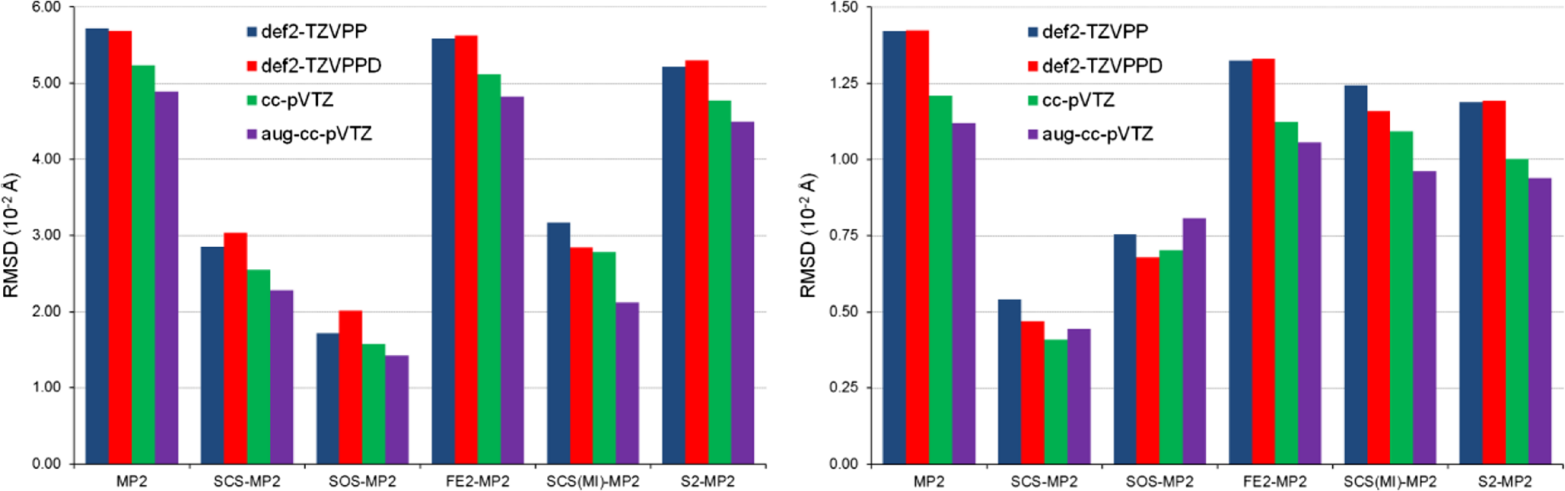
RMSD for the π-complex of SnH_2_ with benzene (*left*) and the σ-complex of SnH_2_ with pyridine (*right*) obtained by MP2 and its variants in combination with four basis sets

Let us now turn our attention to the results of the assessment of six MP2-type methods for predicting the geometries of SnH_2_-benzene and SnH_2_-pyridine. The assessment was performed separately for the geometry of both complexes and the quantitative comparison of the geometries optimized by the MP2-type methods with the geometries obtained from CCSD was based on RMSD. The values of RMSD express quantitatively the deviation of the geometries optimized by the MP2-type methods from the CCSD geometries. The lower the value of RMSD for a given MP2-type method is obtained, the smaller the difference in the geometries yielded by this method and by CCSD occurs. The values of RMSD for SnH_2_-benzene and SnH_2_-pyridine are presented graphically in Fig. 2. As can be deduced from the left-hand plot in this figure, the SOS-MP2 method yields the lowest values of RMSD for the π-complex. These values are below 0.02 Å for all four basis sets considered. Larger RMSD values are produced by SCS-MP2 and SCS(MI)-MP2 but the order of these two methods with respect to their RMSD values is dependent on the basis set applied. The SnH_2_-benzene complex optimized by the conventional MP2 method is in the worst agreement with the CCSD geometry. Regarding how the choice of basis set affects the SnH_2_-benzene geometry, it can be seen that the addition of the set of diffuse functions to the cc-pVTZ basis set decreases the values of RMSD. On the other hand, the application of the def2-TZVPPD basis set does not always improve the reproduction of the π-complex geometry as compared to the result obtained with the def2-TZVPP basis set. It might result from the fact that the set of diffuse functions in def2-TZVPPD was primarily designed to achieve high accuracy for molecular polarizabilities [64]. The geometry of the SnH_2_-pyridine complex is reproduced best by the SCS-MP2 method. This method is followed by SOS-MP2, irrespective of the basis set employed. The effect of diffuse functions on the geometry of the σ-complex is irregular: the addition of the ‘aug’ set to cc-pVTZ increases the value of RMSD for SCS-MP2 and SOS-MP2 but the remaining MP2-type methods exhibit a decrease in their RMSD on going from cc-pVTZ to aug-cc-pVTZ.

It is noteworthy that the values of RMSD for SnH_2_-pyridine are generally lower than those for SnH_2_-benzene. It indicates that for the σ-complexation the MP2-type methods tend to provide a structural description that is more consistent with that of CCSD than is observed for the π-complexation. For both types of complexation the MP2, FE2-MP2, and S2-MP2 combined with all four basis sets systematically yield structures that differ from the CCSD structures significantly. Of all six MP2-type methods applied here, the conventional one shows the largest values of RMSD, hence the use of any of the five SCS schemes considered in this work leads to a reduction in the RMSD values for both types of complexation. The improvement in geometry predicted by SCS-MP2 over that optimized by MP2 was previously reported for various systems containing gold [67, 68]. It was also suggested only recently that SOS-MP2 seems to be superior to MP2 in calculating geometrical parameters for the complex of benzene with tetracyanoethylene [69]. The best performance of the SCS-MP2 method in reproducing the structure of SnH_2_-pyridine is in line with our previous findings on the robustness of this method for the structural characterization of intermolecular N → Sn coordination in trimethyltin cyanide [70].

The results of the assessment performed using RMSD can also be confirmed by the deviations in the values of the key structural parameters characterizing the π- and σ-complexations. Three structural parameters, namely one interatomic distance (*d*) and two angles (*a*_1_ and *a*_2_), were selected. In consequence of such a selection, the π-complexation in SnH_2_-benzene is described by the Sn-C^*^ distance, the Sn-C^*^-H^*^ valence angle and the angle between the molecular plane of SnH_2_ and the molecular plane of the benzene ring (see Fig. 1, the atoms marked with an asterisk are specified in the figure). In the case of the SnH_2_-pyridine complex, *d* denotes the Sn-N distance, *a*_1_ is the Sn-N-C^*^ valence angle and *a*_2_ specifies the angle between the molecular plane of SnH_2_ and the plane perpendicular to the pyridine ring (a fragment of the perpendicular plane is colored green in the right-hand panel in Fig. 1). The values of the three structural parameters for the SnH_2_-benzene and SnH_2_-pyridine complexes optimized using six MP2-type methods combined with the aug-cc-pVTZ basis set are listed in Table 2 (additional results obtained using the remaining three basis sets can be found in Tables S1-S3, see Electronic supplementary material). This table also shows the deviations in the values of the parameters calculated using the MP2-type methods from the reference values computed from the CCSD/aug-cc-pVTZ optimizations. The values of the deviations firmly support the results of the assessment performed using RMSD. Moreover, it is apparent that the conventional MP2 method shortens the *d* distance in both complexes significantly, while the SOS-MP2 method overestimates it slightly. The values of the *a*_1_ angle for the π-complex (*a*_1_ > 90°) indicate that the Sn atom is not positioned exactly over one of the C atoms of benzene but it is shifted toward the center of the benzene ring minimally.

**Table 2:** Selected geometrical parameters (*d*, *a*_1_, and *a*_2_) for the π-complex of SnH_2_ with benzene and for the σ-complex of SnH_2_ with pyridine optimized by six MP2-type methods and CCSD in combination with the aug-cc-pVTZ basis set

Method	SnH_2_-benzene			SnH_2_-pyridine		
	*d*	*a*_1_	*a*_2_	*d*	*a*_1_	*a*_2_
MP2	2.721 (−0.105)	92.39 (2.82)	10.17 (−4.11)	2.313 (−0.022)	119.79 (−0.33)	5.71 (0.10)
SCS-MP2	2.790 (−0.036)	91.40 (1.84)	11.94 (−2.34)	2.336 (0.001)	120.06 (−0.06)	5.74 (0.14)
SOS-MP2	2.829 (0.003)	90.90 (1.33)	12.80 (−1.47)	2.347 (0.012)	120.19 (0.07)	5.77 (0.16)
FE2-MP2	2.726 (−0.100)	92.44 (2.87)	10.23 (−4.04)	2.316 (−0.019)	119.87 (−0.26)	5.85 (0.24)
SCS(MI)-MP2	2.771 (−0.055)	90.79 (1.23)	11.99 (−2.29)	2.325 (−0.010)	119.62 (−0.50)	4.83 (−0.78)
S2-MP2	2.734 (−0.092)	92.32 (2.75)	10.46 (−3.82)	2.318 (−0.017)	119.90 (−0.22)	5.86 (0.25)
CCSD	2.826	89.57	14.27	2.335	120.12	5.61

For the MP2-type methods the deviations of the three parameters from the values calculated using CCSD/aug-cc-pVTZ are shown in parentheses

Distances in Å, angles in °. Each deviation is calculated as the difference between a value obtained from a given MP2-type method and the corresponding reference result

For the SnH_2_-benzene and SnH_2_-pyridine complexes optimized by six MP2-type methods, the interaction energy between SnH_2_ and benzene/pyridine was calculated using the same methodologies as those applied to the optimizations. Both uncorrected (*E*_int_) and counterpoise-corrected (*E*_int_^CP^) interaction energies were considered. In order to provide the reference values of *E*_int_ and *E*_int_^CP^ for each basis set, the CCSD(T) calculations were carried out for the complexes optimized at the CCSD level of theory. The values of *E*_int_ and *E*_int_^CP^ calculated using the aug-cc-pVTZ basis set are presented in Table 3 (additional results obtained using the remaining three basis sets can be found in Tables S4-S6, see Electronic supplementary material). The deviations in the interaction energies predicted by the MP2-type methods from the reference CCSD(T) values are also tabulated. Perusing Table 3 leads to several findings. First, the interaction in the σ-complex is about twice as strong as that in the π-complex. Second, the comparison of *E*_int_ with *E*_int_^CP^ reveals that the counterpoise correction for the BSSE in the interaction energies for both complexes is large and its magnitude can reach several kcal mol^-1^ (the maximal correction of 4.90 kcal mol^-1^ was found for the σ-complex at the FE2-MP2/aug-cc-pVTZ level of theory). The addition of the set of diffuse functions to both def2-TZVPP and cc-pVTZ results in higher values of the counterpoise correction for the BSSE, although such an addition formally increases the completeness of these basis sets and a decrease in the BSSE could actually be expected. However, when the diffuse functions of def2-TZVPPD and aug-cc-pVTZ are centered on one molecular moiety, they overlap to a certain extent the centers on the other moiety (e.g., in the π-complex the diffuse functions ascribed to SnH_2_ may overlap the centers of the benzene molecule), which causes an increase in the counterpoise correction for the BSSE [71]. The increase of BSSE by adding diffuse functions was also reported for the MP2/cc-pVDZ and MP2/aug-cc-pVDZ calculations of interaction energy in benzene dimers [72]. Third, the smallest deviations in *E*_int_^CP^ are found for SCS-MP2 irrespective of the basis set employed. The SCS(MI)-MP2 method produces the lowest values of the deviations in *E*_int_ but only when combined with the aug-cc-pVTZ basis set. For the remaining three basis sets the SCS-MP2 method affords the best agreement with the CCSD(T) values of *E*_int_. The conventional MP2 method, by contrast, always yields the most significant deviations in both *E*_int_ and *E*_int_^CP^, and this method severely overestimates the strength of the interaction between SnH_2_ and benzene/pyridine. Too negative values of *E*_int_ and *E*_int_^CP^ calculated by MP2 result from the application of the uncoupled Hartree-Fock dispersion energy that is known to be overestimated in MP2. The overestimation of the interaction in SnH_2_-benzene and SnH_2_-pyridine by the MP2 method is also accompanied by the shortening of the distance between SnH_2_ and benzene/pyridine, as it was illustrated by the *d* parameter and its deviation in Table 2. The poor performance of the conventional MP2 method was previously reported for predicting interaction energy in the complexes of transition metals with various ligands [67, 68], including benzene [73]. Two of these studies [67, 68] also showed that the SCS-MP2 method exhibits better performance in calculating the interaction energy than the conventional MP2 method, which is in line with our observation that SCS-MP2 is able to correct the MP2 overestimation of the interaction energy in SnH_2_-benzene and SnH_2_-pyridine.

**Table 3:** *E*_int_ and *E*_int_^CP^ obtained by six MP2-type methods and CCSD(T) in combination with the aug-cc-pVTZ basis set

Method	SnH_2_-benzene		SnH_2_-pyridine	
	*E*_int_	*E*_int_^CP^	*E*_int_	*E*_int_^CP^
MP2	−16.02 (−3.23)	−11.51 (−2.42)	−30.18 (−2.45)	−25.42 (−2.05)
SCS-MP2	−12.74 (0.05)	−8.83 (0.25)	−27.18 (0.55)	−22.83 (0.53)
SOS-MP2	−11.22 (1.57)	−7.60 (1.48)	−25.71 (2.02)	−21.57 (1.80)
FE2-MP2	−15.84 (−3.05)	−11.20 (−2.12)	−29.99 (−2.26)	−25.09 (−1.72)
SCS(MI)-MP2	−12.99 (−0.20)	−10.13 (−1.05)	−27.61 (0.11)	−24.42 (−1.06)
S2-MP2	−15.41 (−2.62)	−10.84 (−1.76)	−29.60 (−1.88)	−24.75 (−1.38)
CCSD(T)	−12.79	−9.08	−27.73	−23.36

For the MP2-type methods the deviations of their two interaction energies from the values calculated using CCSD(T)/aug-cc-pVTZ are shown in parentheses

All values in kcal mol^-1^

**Fig. 3:**
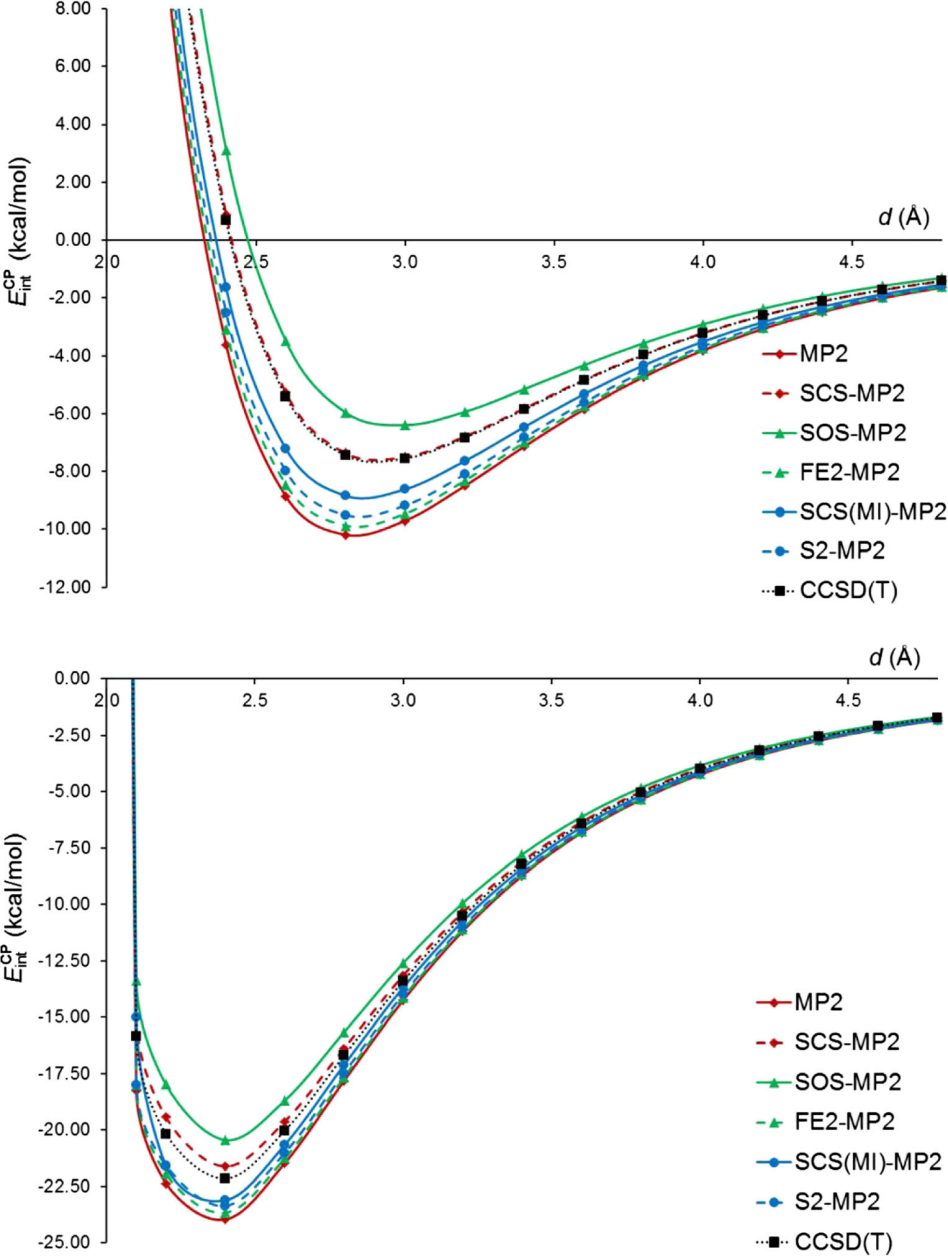
*E*_int_^CP^ for the π-complex of SnH_2_ with benzene (*top*) and the σ-complex of SnH_2_ with pyridine (*bottom*) as a function of the geometrical parameter *d*. The MP2-type and CCSD(T) methods were combined with the aug-cc-pVTZ basis set

Aside from the determination of *E*_int_ and *E*_int_^CP^ for SnH_2_-benzene and SnH_2_-pyridine in their geometries optimized by different methods, it is essential to evaluate the performance of the MP2-type methods for the fixed geometries of the complexes. However, instead of inspecting a single geometry of each complex, we decided to do a survey of a set of geometries with a wide range of the values of the *d* parameter. In each complex the geometries of the SnH_2_ and the benzene/pyridine moieties were fixed and a number of single-point calculations using the MP2-type methods and CCSD(T) were carried out for the *d* parameter in the range from 2.0 to 5.0 Å. It should be stressed that for each value of the *d* parameter the same geometry of a given complex was used by all the methods. The resulting set of points, whose coordinates are interaction energies and the corresponding *d* values, can be presented graphically in the form of an interaction energy curve. For the purposes of illustration, the curves of *E*_int_^CP^ for the MP2-type and CCSD(T) methods combined with the aug-cc-pVTZ basis set are plotted in Fig. 3. The inspection of this figure clearly shows that the SCS-MP2 method provides best agreements with the CCSD(T) results. The SCS-MP2 curve for the π-complex almost perfectly matches the reference curve of *E*_int_^CP^, whereas the SCS-MP2 curve for the σ-complex is slightly above the CCSD(T) curve. The SOS-MP2 curve for the σ-complex is even higher than the SCS-MP2 one and the same can be noticed for the π-complex. By contrast, the curves computed with FE2-MP2, SCS(MI)-MP2, S2-MP2, and MP2 are below the CCSD(T) curve in the minimum region as well as for larger *d* in both complexes. The behavior of the MP2-type methods for larger *d* values may be important for investigating the interaction between aromatic molecules and stannylenes containing a bulky substituent. For instance, it is known that some N-heterocyclic stannylenes exhibit Sn-aryl contacts of 3.51 Å [74]. Our observations for the interaction energy curves of SnH_2_-benzene are similar to those made by Takatani and Sherrill [75] for interaction energy curves in the methane-benzene and H_2_S-benzene complexes. Authors noticed that SCS-MP2 offered striking improvements over the conventional MP2 method for the reproduction of the CCSD(T) interaction energy curves of these two complexes. For the H_2_S-benzene complex the SCS-MP2 method underestimated, however, interaction energies compared to CCSD(T).

In order to assess quantitatively the performance of the MP2-type methods in reproducing the CCSD(T) curves of *E*_int_ and *E*_int_^CP^ for SnH_2_-benzene and SnH_2_-pyridine, a set of 13 points in the minimum region and also for larger *d* values was selected. For the set of points obtained with each MP2-type method, the mean signed error (MSE) and the root mean square error (RMSE) with respect to the reference CCSD(T) data were calculated (more details of the MSE and RMSE calculations can be found in section S1, see Electronic supplementary material). Table 4 presents the values of MSE and RMSE in *E*_int_ and *E*_int_^CP^ computed with six MP2-type methods in combination with the aug-cc-pVTZ basis set (results for the remaining three basis sets are gathered in Tables S7-S9, see Electronic supplementary material). The conclusions drawn from the visual inspection of Fig. 3 are confirmed quantitatively by the results in Table 4. The errors for SCS-MP2 are up to two orders of magnitude lower than the errors for the other MP2-type methods. Moreover, the performance of SCS-MP2 is similar in reproducing the curves of both *E*_int_ and *E*_int_^CP^. These findings are valid for all four basis sets.

**Table 4:** MSE and RMSE for *E*_int_ and *E*_int_^CP^ across the interaction energy curves for the SnH_2_-benzene and SnH_2_-pyridine complexes

Method	SnH_2_-benzene				SnH_2_-pyridine			
	*E*_int_		*E*_int_^CP^		*E*_int_		*E*_int_^CP^	
	MSE	RMSE	MSE	RMSE	MSE	RMSE	MSE	RMSE
MP2	−1.64	2.14	−1.48	1.95	−0.89	1.16	−0.77	1.01
SCS-MP2	0.04	0.07	0.05	0.08	0.21	0.31	0.21	0.31
SOS-MP2	0.88	1.16	0.81	1.08	0.77	1.04	0.70	0.96
FE2-MP2	−1.55	2.01	−1.31	1.72	−0.83	1.07	−0.65	0.84
SCS(MI)-MP2	−0.13	0.26	−0.75	1.01	0.14	0.16	−0.36	0.54
S2-MP2	−1.33	1.73	−1.11	1.46	−0.69	0.89	−0.52	0.67

The errors are calculated for the energies obtained from MP2 and its variants combined with the aug-cc-pVTZ basis set with respect to the CCSD(T)/aug-cc-pVTZ energies

All values in kcal mol^-1^

### Assessment of DFT methods

**Fig. 4:**
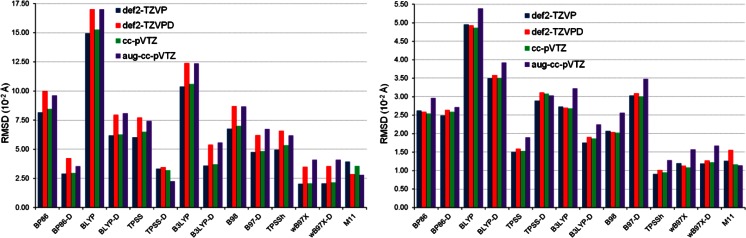
RMSD for the π-complex of SnH_2_ with benzene (*left*) and the σ-complex of SnH_2_ with pyridine (*right*) obtained by 14 DFT methods in combination with four basis sets

Now we turn our attention to the assessment of the performances of 14 DFT methods and this assessment will be carried out using the same evaluation procedures as those applied to the MP2-type methods. The first evaluation procedure involves calculating RMSD for the DFT-optimized structures with respect to the geometries of the SnH_2_-benzene and SnH_2_-pyridine complexes optimized using the reference CCSD method. The resulting values of RMSD are presented graphically in Fig. 4. In order to obtain each RMSD value, a pair of structures optimized using the same basis set was considered. However, as mentioned in the Computational details section, the structures optimized by DFT in conjunction with def2-TZVP and def2-TZVPD were referenced to the structures computed by CCSD/def2-TZVPP and CCSD/def2-TZVPPD, respectively. A quick glance at Fig. 4 reveals that the values of RMSD for the σ-complex are generally much smaller than the RMSD values for the π-complex. The same was observed for the MP2-type methods in Fig. 2. Hence, providing a reliable structural description for the π-complexation between SnH_2_ and benzene is a real challenge both for the MP2-type methods and for the DFT methods.

A detailed analysis of Fig. 4 indicates that two modern standard density functionals, namely ωB97X and M11, yield the π-complex geometries that are in good agreement with the reference structure. These density functionals also perform well in reproducing the CCSD geometry of the σ-complex but additionally the list of the density functionals that are the most suitable for this complex can be extended by the TPSSh density functional. The B3LYP hybrid density functional exhibits disappointing performance in reproducing the structures of both complexes. It is rather not surprising because the poor performance of this functional was detected for the structures of transition-metal complexes from the first [76, 77] and subsequent rows [7, 8]. The comparative study performed by Bühl and Kabrede [76] also showed that B3LYP was outperformed by BP86, TPSS, and TPSSh when equilibrium geometries for a diverse set of 32 metal complexes were taken into consideration. Our results agree with the aforementioned findings. Our observation on the good performance of RSH density functionals seems to extend the findings of two previous benchmark studies [7, 78] in which the ωB97X-D density functional emerged as the most suitable functional for predicting geometries of transition-metal complexes.

It is crucial to discuss the effect of the dispersion correction incorporated into the DFT methods on the reproduction of the geometries of SnH_2_-benzene and SnH_2_-pyridine. The comparison of the RMSD values obtained from standard density functionals with the RMSD values yielded by the corresponding dispersion-corrected density functionals indicates that it is difficult to find any systematic change in all these RMSD values. Moreover, even for a single complex the effect of the dispersion correction turns out to be erratic. For the σ-complex the inclusion of the dispersion correction in BLYP and B3LYP leads to an improvement in the performance of these two density functionals. The reverse situation can be seen for TPSS and B98. The reproduction of the σ-complex geometry by BP86-D and ωB97X-D is hardly affected by the presence of the dispersion correction. In the case of the π-complex, the density functionals belonging to non-RSH generations benefit greatly from the addition of the dispersion correction. In consequence, such dispersion-corrected density functionals as BP86-D and TPSS-D are capable of providing the RMSD values that are very close to those obtained by the RSH density functionals. The accuracy of the π-complex geometry optimized by ωB97X is practically insensitive to the dispersion correction. The absence of any systematic change in the RMSD values obtained by the dispersion-corrected density functionals for both complexes may be due to the fact that the dispersion correction used in this work is a simple empirical correction that covers no system dependence for its damping function and coefficients [58]. Such an absence may, to some extent, illustrate differences between the π-complexation and the σ-complexation (inspecting the nature of such possible differences in detail is, however, beyond the scope of this work). The findings presented in this paragraph are in qualitative agreement with the results of the very recent DFT benchmark by Weymuth et al. [79]. These authors observed a relatively significant influence of dispersion corrections on structures of transition-metal complexes and, what is more important, the structures optimized using different dispersion-corrected density functionals deviated strongly from each other.

The ranking of the density functionals ordered with respect to their RMSD values is rather insensitive to the basis set applied. However, the RMSD value computed with a given density functional and with a given basis set may differ quite noticeably from the RMSD value calculated using the same density functional but combined with a different basis set. In general, augmenting def2-TZVP or cc-pVTZ with their diffuse functions leads to an increase in the values of RMSD for the majority of the density functionals considered. M11 presents an exceptional behavior for Dunning’s basis set and its lowest RMSD values can be found for its combination with the aug-cc-pVTZ basis set.

Since the evaluation procedure utilizing RMSD was applied to the assessment of both the MP2-type and the DFT methods, it is interesting to inspect whether the former can be an alternative to the latter for reproducing the structures of the SnH_2_-benzene and SnH_2_-pyridine complexes. If the RMSD values shown in Fig. 2 are juxtaposed with those in Fig. 4, it is evident that the best MP2-type methods (namely, SOS-MP2 and SCS-MP2) outperform the best DFT methods (e.g., ωB97X). More specifically, the latter produce the RMSD values approximately twice as large as the RMSD values calculated with the former methods. It implies that, of all methods considered, SOS-MP2 and SCS-MP2 are the most suitable for reproducing the reference CCSD geometries of SnH_2_-benzene and SnH_2_-pyridine. Another important issue that deserves noting here is the assessment of the conventional MP2 method by comparing with the performance of the RSH density functionals. Such a comparison reveals that the performance of MP2 in reproducing the geometry of the π-complex is far from acceptable. On the other hand, the conventional MP2 method provides the structural description of the σ-complexation that is comparable to that yielded by the RSH density functionals. The inferior performance of MP2 in comparison to ωB97X and ωB97X-D was reported by Kulkarni and Truhlar in their comparative study of structural parameters in Ru complexes [7].

The values of three characteristic structural parameters describing the interaction between SnH_2_ and benzene/pyridine in the SnH_2_-benzene and SnH_2_-pyridine complexes are listed in Table 5. The tabulated values were obtained from the DFT calculations using the aug-cc-pVTZ basis set (results for the remaining three basis sets can be found in Tables S10-S12, see Electronic supplementary material). The deviations in *d*, *a*_1_, and *a*_2_ calculated by 14 density functionals from the corresponding CCSD/aug-cc-pVTZ results are also presented in Table 5. The analysis of these deviations and the findings made in terms of RMSD in the previous paragraphs are broadly similar and they lead to identical conclusions on the performance of individual density functionals. Besides, the results shown in Table 5 allow us to gain a more detailed structural picture of complexation in SnH_2_-benzene and SnH_2_-pyridine. On the basis of many positive deviations in *d*, it is apparent that the majority of the DFT methods overestimate the *d* distance in both complexes. All DFT methods except M11 predict that the SnH_2_ molecule is moved slightly outward the carbon ring of the benzene molecule (as is indicated by *a*_1_ < 90°) and the molecular plane of SnH_2_ is inclined toward the benzene plane to a greater extent than it is observed in the π-complex optimized by CCSD. The deviations in *a*_1_ and *a*_2_ for the σ-complex do not show clear regularities but they are generally much smaller than those obtained for the π-complex.

**Table 5:** Selected geometrical parameters (*d*, *a*_1_, and *a*_2_) for the π-complex of SnH_2_ with benzene and for the σ-complex of SnH_2_ with pyridine optimized by 14 DFT methods in combination with the aug-cc-pVTZ basis set

Method	SnH_2_-benzene			SnH_2_-pyridine		
	*d*	*a*_1_	*a*_2_	*d*	*a*_1_	*a*_2_
BP86	2.838 (0.012)	85.07 (−4.50)	19.00 (4.72)	2.391 (0.056)	119.91 (−0.22)	5.63 (0.02)
BP86-D	2.834 (0.007)	88.16 (−1.40)	16.67 (2.39)	2.381 (0.046)	120.85 (0.73)	8.26 (2.65)
BLYP	2.987 (0.161)	81.23 (−8.34)	22.72 (8.45)	2.444 (0.109)	120.04 (−0.08)	5.36 (−0.25)
BLYP-D	2.948 (0.122)	85.84 (−3.73)	18.62 (4.35)	2.426 (0.091)	120.92 (0.80)	7.99 (2.39)
TPSS	2.810 (−0.016)	86.04 (−3.53)	19.20 (4.92)	2.376 (0.041)	120.38 (0.26)	6.70 (1.09)
TPSS-D	2.805 (−0.021)	89.19 (−0.37)	16.58 (2.31)	2.369 (0.034)	121.39 (1.26)	9.40 (3.79)
B3LYP	2.949 (0.123)	83.29 (−6.28)	20.87 (6.59)	2.409 (0.074)	119.96 (−0.17)	5.32 (−0.29)
B3LYP-D	2.926 (0.100)	86.75 (−2.82)	17.82 (3.54)	2.399 (0.064)	120.77 (0.64)	7.61 (2.01)
B98	2.902 (0.076)	85.39 (−4.18)	18.83 (4.56)	2.395 (0.060)	119.98 (−0.14)	5.50 (−0.11)
B97-D	2.960 (0.134)	87.24 (−2.33)	16.47 (2.19)	2.421 (0.086)	120.59 (0.47)	7.18 (1.57)
TPSSh	2.808 (−0.018)	86.61 (−2.96)	18.44 (4.17)	2.368 (0.033)	120.29 (0.17)	6.46 (0.85)
ωB97X	2.919 (0.093)	88.19 (−1.38)	16.11 (1.84)	2.380 (0.045)	120.24 (0.12)	5.81 (0.21)
ωB97X-D	2.923 (0.097)	88.33 (−1.24)	16.54 (2.27)	2.384 (0.049)	120.49 (0.37)	6.44 (0.83)
M11	2.855 (0.029)	90.68 (1.11)	13.03 (−1.24)	2.364 (0.029)	120.56 (0.44)	6.72 (1.11)
CCSD	2.826	89.57	14.27	2.335	120.12	5.61

The deviations of the three parameters from the values calculated using CCSD/aug-cc-pVTZ are shown in parentheses. In the last row there are the CCSD/aug-cc-pVTZ results, repeated after Table 2

Distances in Å, angles in °

Next, for the SnH_2_-benzene and SnH_2_-pyridine complexes optimized by 14 DFT methods, the uncorrected and counterpoise-corrected interaction energies between SnH_2_ and benzene/pyridine were calculated using these methods. The values of *E*_int_ and *E*_int_^CP^ obtained from the DFT methods in conjunction with the aug-cc-pVTZ basis set are listed in Table 6 (results for the remaining three basis sets are gathered in Tables S13-S15, see Electronic supplementary material). In order to evaluate the performance of the DFT methods in predicting *E*_int_ and *E*_int_^CP^, Table 6 also presents the deviations of the DFT results from the CCSD(T)/aug-cc-pVTZ interaction energies calculated for the complex geometries optimized at the CCSD/aug-cc-pVTZ level of theory. As it can be seen in this table, the TPSS-D density functional yields the smallest deviations in *E*_int_, whereas ωB97X and M11 give the best agreements for *E*_int_^CP^ in SnH_2_-benzene and SnH_2_-pyridine, respectively. This suggests that there is no single density functional that is the most accurate for *E*_int_ and *E*_int_^CP^ in both complexes. As a matter of fact, the ranking of the DFT methods ordered with respect to their performance for *E*_int_ is not exactly the same as the ranking compiled for *E*_int_^CP^. Moreover, the performance of the DFT methods turns out to be rather sensitive to the choice of basis set. Nevertheless, some regularities in the calculated interaction energies and in their deviations from the CCSD(T) results can be found. The BLYP density functional produces the largest deviations in *E*_int_, irrespective of the basis set applied. The performance of this functional in reproducing *E*_int_^CP^ is also very poor but it is not necessarily associated with the largest deviations for all basis sets. The poor performance of BLYP seems to be due to the LYP correlation [80] incorporated in this density functional. It was previously reported that density functionals with the LYP correlation failed in describing some coinage metal systems [81–83]. The LYP correlation does not satisfy the correct uniform electron gas limit, which was the reason for the poor performance of the BLYP density functional in modeling coinage metal systems and most probably in our case as well. B3LYP outperforms BLYP but the performance of the former in reproducing *E*_int_ and *E*_int_^CP^ is also unsatisfactory, which again can be explained by the presence of the LYP correlation. In addition, it is known that the B3LYP density functional tends to overestimate the absolute exchange and correlation energies of molecules containing metals compared to accurate coupled cluster calculations [84]. If BLYP and B3LYP are not under consideration, the remaining GGA and GH GGA density functionals (that is, BP86 and B98) exhibit larger deviations in *E*_int_ and *E*_int_^CP^ for both complexes than the meta-GGA and GH meta-GGA density functionals (that is, TPSS and TPSSh). The deviations yielded by TPSS are much the same as those obtained by TPSSh for all four basis sets. The accuracies exhibited by BP86 and TPSS are in accord with the ‘Jacob’s ladder’ classification of DFT methods [85]. Moving up from the second rung (GGA) to the third rung (meta-GGA), indeed, leads to the improvement in the predicted values of *E*_int_ and *E*_int_^CP^. The performance of the RSH density functionals in reproducing *E*_int_ is good in the sense that their deviations are lower than those of meta-GGA and GH meta-GGA for both complexes and for all basis sets. In the case of *E*_int_^CP^, the performance of ωB97X and M11 is, however, dependent on the basis set employed. Only the most extended basis set, namely aug-cc-pVTZ, allows these two density functionals to predict *E*_int_^CP^ with great accuracy. Our conclusions are in line with the results of a previous benchmark study devoted to the performance of density functional for the bond energetics of 3*d* transition-metal compounds [77]. For such compounds B3LYP is outperformed by BP86 and the following functionals are TPSS and TPSSh. The good performance of M11 is not well-established in the literature and even the latest comparative studies of Minnesota density functionals often do not include this functional [86–88]. It has been reported so far that M11 emerges as a very reliable functional for the study of the energetics of sulfate-water clusters [89]. From a more general perspective, it was recently suggested that the introduction of range-separation can fix errors caused by the absence of dispersion corrections in standard density functionals and an improvement in interaction energies calculated by range-separated density functionals was observed for Grubbs catalysts [90]. The improvement can most probably be ascribed to some cancellation of errors as range-separation selectively turns off the gradient-corrected part of density functionals and it leads to LDA-type overbinding [90]. The results presented in Table 6 confirm that the RSH density functionals predict stronger interaction between SnH_2_ and benzene/pyridine than the GGA density functionals.

**Table 6:** *E*_int_ and *E*_int_^CP^ for the π-complex of SnH_2_ with benzene and for the σ-complex of SnH_2_ with pyridine optimized by 14 DFT methods in combination with the aug-cc-pVTZ basis set

Method	SnH_2_-benzene		SnH_2_-pyridine	
	*E*_int_	*E*_int_^CP^	*E*_int_	*E*_int_^CP^
BP86	−6.63 (6.16)	−6.62 (2.46)	−21.35 (6.38)	−21.15 (2.22)
BP86-D	−12.50 (0.29)	−12.32 (−3.23)	−25.73 (2.00)	−25.52 (−2.16)
BLYP	−4.11 (8.68)	−4.10 (4.98)	−18.40 (9.33)	−18.20 (5.16)
BLYP-D	−10.43 (2.36)	−10.27 (−1.19)	−23.28 (4.45)	−23.07 (0.29)
TPSS	−7.36 (5.42)	−7.23 (1.85)	−22.40 (5.33)	−22.23 (1.14)
TPSS-D	−12.93 (−0.14)	−12.79 (−3.71)	−26.63 (1.10)	−26.46 (−3.09)
B3LYP	−4.92 (7.87)	−4.92 (4.17)	−19.60 (8.13)	−19.43 (3.93)
B3LYP-D	−10.63 (2.16)	−10.49 (−1.40)	−23.94 (3.79)	−23.77 (−0.40)
B98	−6.71 (6.08)	−6.73 (2.36)	−20.98 (6.75)	−20.82 (2.54)
B97-D	−10.93 (1.86)	−10.87 (−1.79)	−23.07 (4.66)	−22.88 (0.48)
TPSSh	−7.38 (5.41)	−7.26 (1.83)	−22.43 (5.30)	−22.28 (1.09)
ωB97X	−9.38 (3.41)	−9.28 (−0.19)	−24.07 (3.66)	−23.83 (−0.46)
ωB97X-D	−9.92 (2.87)	−9.86 (−0.77)	−23.75 (3.98)	−23.56 (−0.19)
M11	−10.31 (2.48)	−9.77 (−0.68)	−23.79 (3.94)	−23.40 (−0.04)
CCSD(T)	−12.79	−9.08	−27.73	−23.36

The deviations of the two interaction energies from the values calculated using CCSD(T)/aug-cc-pVTZ are shown in parentheses. In the last row there are the CCSD(T)/aug-cc-pVTZ results, repeated after Table 3

All values in kcal mol^-1^

As can be seen in Table 6, the effect of the dispersion correction on the values of *E*_int_ and *E*_int_^CP^ is rather erratic. On the one hand, the addition of the dispersion correction decreases the deviations in *E*_int_ for all dispersion-corrected density functionals except for ωB97X-D in SnH_2_-pyridine. Such an effect is valid for all four basis sets. On the other hand, the effect of the dispersion correction on the values of *E*_int_^CP^ is heavily dependent on the density functional, basis set and kind of complex. The lack of a uniform improvement of density functionals by dispersion corrections was also detected for the interaction between gold and unsaturated aliphatic hydrocarbons [68]. We observe, however, that the addition of the D correction to BLYP and B3LYP consistently improves their *E*_int_^CP^ values.

Another important issue is how the choice of basis set affects the values of *E*_int_ and *E*_int_^CP^. For each DFT method, its values of *E*_int_ obtained using the cc-pVTZ basis set are fairly similar to those calculated with def2-TZVP. The same can be observed for *E*_int_^CP^. The differences between interaction energies computed with these two basis sets do not exceed 0.65 kcal mol^-1^. The presence of diffuse functions in def2-TZVPD and aug-cc-pVTZ often increases the deviations in *E*_int_ and *E*_int_^CP^ compared to the corresponding deviations yielded by the DFT methods in conjunction with def2-TZVP and cc-pVTZ. However, combining the dispersion-corrected or RSH density functionals with the diffuse-augmented basis sets leads to the *E*_int_^CP^ values that are in better agreement with the reference results.

It is essential to estimate the accuracy of the DFT methods for reproducing *E*_int_ and *E*_int_^CP^ from a more global viewpoint, covering the variations in *E*_int_ and *E*_int_^CP^ obtained by both the DFT and the MP2-type methods with respect to the reference CCSD(T) data. A closer inspection of the variations in *E*_int_ calculated using the DFT and MP2-type methods reveals that the *E*_int_ values yielded by the former methods differ more significantly from the reference values (compare the *E*_int_ values and their variations in Tables 3 and 6). It is largely due to the fact that the *E*_int_ values calculated using the MP2-type methods are affected by the BSSE to the extent that is similar to that observed for the CCSD(T) values. By contrast, the DFT methods produce the *E*_int_ values that are hardly influenced by the BSSE (compare *E*_int_ and *E*_int_^CP^ in Table 6). If one collates the variations in *E*_int_^CP^ produced by the DFT methods with the variations in *E*_int_^CP^ predicted by SCS-MP2, it is clear that the SCS-MP2 method generally outperforms the DFT methods in reproducing the CCSD(T) values of *E*_int_^CP^. The variations in *E*_int_^CP^ yielded by the DFT methods are heavily dependent on the choice of basis set and, therefore, it is impossible to find a single density functional that combined with all considered basis sets always achieves accuracy that is comparable to that of SCS-MP2. This finding stands in marked contrast to the results obtained for dissociation energies of gold complexes whose dissociation energies were predicted by the best performing DFT methods more accurately than by SCS-MP2 [68].

**Fig. 5:**
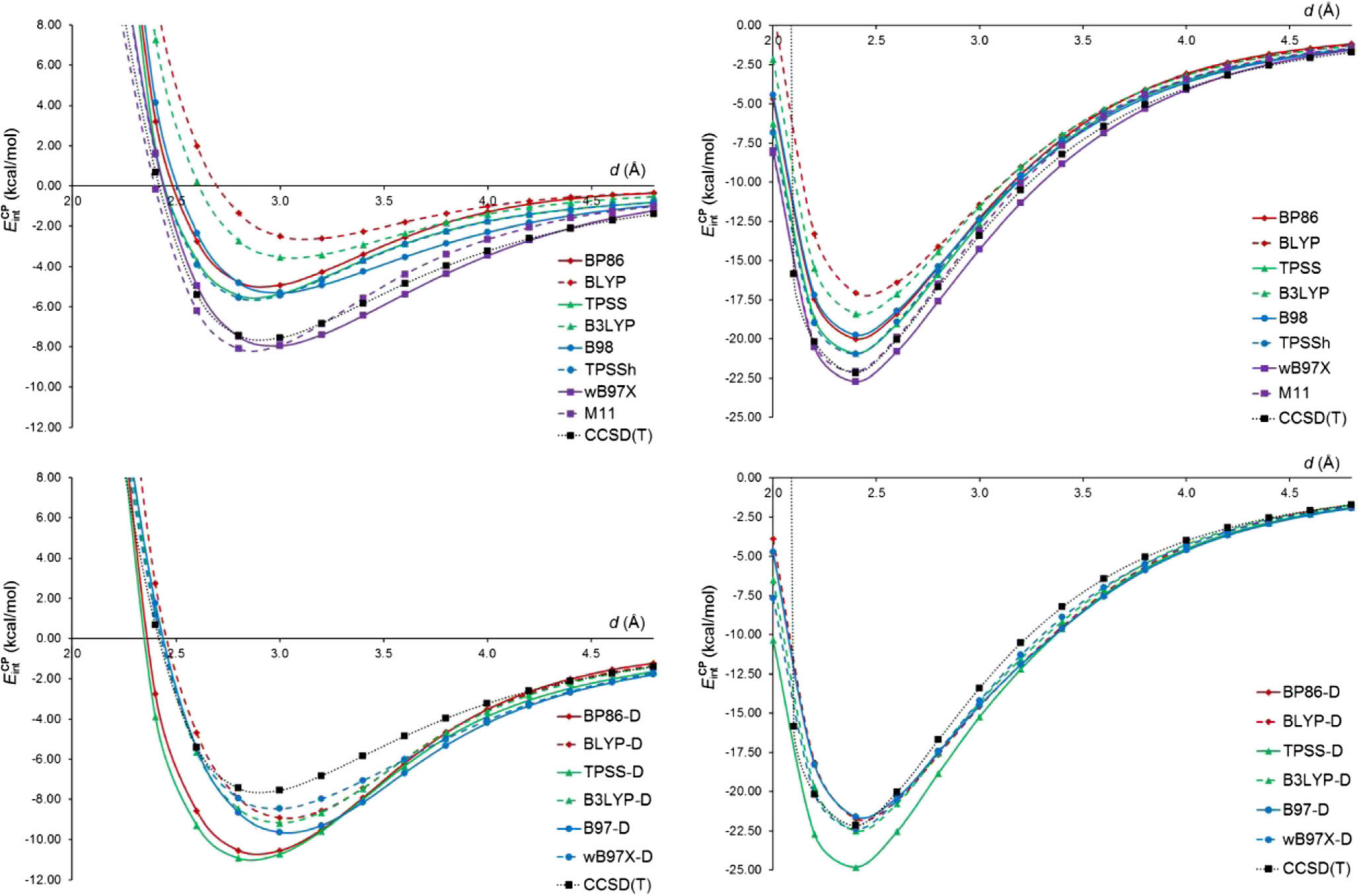
*E*_int_^CP^ for the π-complex of SnH_2_ with benzene (*left*) and the σ-complex of SnH_2_ with pyridine (*right*) as a function of the geometrical parameter *d*. Fourteen DFT methods were combined with the aug-cc-pVTZ basis set. The *E*_int_^CP^ curves obtained at the CCSD(T)/aug-cc-pVTZ level are also shown for comparison

As the next stage of the assessment of the DFT methods, the ability of these methods to reproduce the interaction energies as a function of the *d* parameter in the SnH_2_-benzene and SnH_2_-pyridine complexes has been studied. It allows us to exclude the effect of different complex geometries optimized by various methods from the analysis of *E*_int_ and *E*_int_^CP^. It is so because for each value of the *d* parameter the same complex geometry was used by both the DFT methods and CCSD(T) for calculating *E*_int_ and *E*_int_^CP^. Figure 5 presents the exemplary plots of *E*_int_^CP^ as a function of *d* in SnH_2_-benzene and SnH_2_-pyridine for 14 DFT methods combined with the aug-cc-pVTZ basis set. For clarity, the DFT methods are divided into two groups, without and with the dispersion correction. It is evident from Fig. 5 that BP86, BLYP, TPSS, B3LYP, B98, and TPSSh underestimate the strength of the interaction in the π-complex in the whole range of *d* values. By contrast, ωB97X and M11 slightly overestimate the binding around the minimum region in this complex but their curves of *E*_int_^CP^ are generally much closer to the CCSD(T) curve than those obtained by six non-RSH standard density functionals. The comparison of two plots on the left-hand side in Fig. 5 reveals that the addition of the dispersion correction systematically lowers *E*_int_^CP^ toward more negative values. However, it becomes apparent that the addition of the dispersion correction not only compensates for the *E*_int_^CP^ underestimation typical of the non-RSH standard density functionals but, unfortunately, leads to some overestimation of *E*_int_^CP^ in the minimum region. The TPSS-D density functional demonstrates the most significant overestimation in this region. A severe overestimation of interaction energies by TPSS in conjunction with Grimme’s dispersion correction was also detected for the complexes of benzene with main group [44] and transition metal atoms [73]. The dispersion-corrected density functionals provide a much better description of the interaction for very large *d* values (*d* > 4.5 Å) in the π-complex. Among the density functionals without the dispersion correction, ωB97X and M11 produce the *E*_int_^CP^ curves that are the closest to the CCSD(T) one also for the σ-complex. With the values of *d* ranging in the 3–4 Å interval, ωB97X overestimates the interaction between SnH_2_ and pyridine slightly, whereas M11 exhibits the underestimation of *E*_int_^CP^. It is rather of secondary importance to practical calculations that the slope of the *E*_int_^CP^ curves for small *d* values (*d* < 2.1 Å) is significantly less steep for the DFT methods than for CCSD(T). The *E*_int_^CP^ curves of the DFT methods incorporating the dispersion correction differ from the CCSD(T) curve of *E*_int_^CP^ marginally. Only the TPSS-D density functional exhibits an exceptional behavior in the minimum region where a significant overbinding of SnH_2_ by pyridine is predicted with use of this functional. The comparison of the bottom plots in Fig. 5 also indicates that if the dispersion correction is introduced to BP86, BLYP, B3LYP, B98, and ωB97X, then the reproduction of the reference *E*_int_^CP^ curve is more effective for the σ-complex than for the π-complex.

In order to facilitate the quantitative evaluation of the performance of the DFT methods in reproducing the CCSD(T) curves of *E*_int_ and *E*_int_^CP^ in SnH_2_-benzene and SnH_2_-pyridine, the values of MSE and RMSE in *E*_int_ and *E*_int_^CP^ for the curves obtained by each density functional were determined. Table 7 gathers the values of MSE and RMSE in *E*_int_ and *E*_int_^CP^ calculated by 14 DFT methods in conjunction with the aug-cc-pVTZ basis set (results for the remaining three basis sets are given in Tables S16-S18, see Electronic supplementary material). A survey of the tabulated results clearly shows that the performance of the DFT methods is largely affected by the presence of counterpoise correction in the interaction energy, the type of complexation and the choice of basis set. We will discuss the errors in *E*_int_ first. When the def2-TZVP and cc-pVTZ basis sets are taken into consideration, the CCSD(T) curves of *E*_int_ are reproduced best by the M11 density functional (see the respective values of RMSE in Tables S16 and S18). The augmentation of these basis sets with their sets of diffuse functions leads to a loss in accuracy for M11 and the combinations of BP86-D and TPSS-D with def2-TZVPD or aug-cc-pVTZ produce the *E*_int_ curves that are most consistent with the reference curves. The values of RMSE for M11/def2-TZVPD and M11/aug-cc-pVTZ indicate that the performance of M11 is close to that of BLYP-D, B3LYP-D, and B97-D. These dispersion-corrected density functionals perform considerably worse than BP86-D and TPSS-D. Although the addition of the dispersion correction to ωB97X may result in a modest increase in RMSE, the density functionals investigated here generally benefit from the inclusion of such a correction. The values of MSE in *E*_int_ in Table 7 are all positive, which means that the DFT curves tend to be placed above the CCSD(T) curve on the axis of *E*_int_. For the remaining basis sets there is, however, no such regularity in the position of the DFT curves. In the case of *E*_int_^CP^, the performance of the DFT methods in reproducing the CCSD(T) curves changes with the type of complexation. For the π-complex, the ωB97X density functional affords the best agreement with the CCSD(T) curve of *E*_int_^CP^. ωB97X is followed by M11 irrespective of the basis set employed. Among the non-RSH standard density functionals, TPSS and TPSSh seem to be the most suitable for the reliable determination of the *E*_int_^CP^ curve in the π-complex. Slightly larger values of RMSD occur for such dispersion-corrected density functionals as BP86-D, BLYP-D, B3LYP-D, and B97-D. The reference curve of *E*_int_^CP^ in the σ-complex is mimicked best by M11 if this density functional is used together with aug-cc-pVTZ. The application of the other basis sets favors TPSS and TPSSh. As indicated by the values of MSE in *E*_int_^CP^, these two functionals have a tendency to compute a weaker interaction between SnH_2_ and pyridine across the investigated range of the *d* parameter in comparison to the interaction predicted by CCSD(T). Introducing the dispersion correction to BLYP and B3LYP always leads to a substantial improvement in reproducing the reference *E*_int_^CP^ curve for the σ-complex. However, the other density functionals do not necessarily exhibit a decrease in RMSE and a change of MSE to zero when they are used with the dispersion correction. In consequence, one can postulate that the application of the dispersion correction to the density functional that as such yields good agreement with the reference *E*_int_^CP^ curve for the σ-complex is inefficient.

**Table 7:** MSE and RMSE in *E*_int_ and *E*_int_^CP^ across the interaction energy curves for the SnH_2_-benzene and SnH_2_-pyridine complexes

Method	SnH_2_-benzene				SnH_2_-pyridine			
	*E*_int_		*E*_int_^CP^		*E*_int_		*E*_int_^CP^	
	MSE	RMSE	MSE	RMSE	MSE	RMSE	MSE	RMSE
BP86	4.26	4.72	2.11	2.18	2.97	3.53	1.24	1.37
BP86-D	0.66	0.91	−1.49	2.04	0.76	1.16	−0.97	1.25
BLYP	5.92	7.11	3.76	4.45	3.86	5.01	2.13	2.85
BLYP-D	1.81	2.83	−0.35	1.06	1.34	2.38	−0.39	0.95
TPSS	3.74	4.17	1.55	1.64	2.48	3.00	0.73	0.80
TPSS-D	0.31	0.48	−1.88	2.39	0.38	0.76	−1.37	1.63
B3LYP	5.15	6.12	2.96	3.44	3.51	4.37	1.76	2.17
B3LYP-D	1.55	2.34	−0.64	0.96	1.30	1.97	−0.44	0.61
B98	3.78	4.55	1.59	1.86	2.76	3.53	1.00	1.32
B97-D	1.14	2.36	−1.03	1.41	1.26	2.39	−0.48	0.97
TPSSh	3.67	4.08	1.48	1.58	2.58	3.04	0.81	0.87
ωB97X	2.05	2.73	−0.09	0.44	1.30	1.77	−0.41	0.54
ωB97X-D	1.53	2.42	−0.63	0.80	1.32	1.93	−0.42	0.49
M11	2.01	2.22	0.08	0.55	2.00	2.28	0.36	0.43

The errors are calculated for the interaction energies obtained from 14 DFT methods combined with the aug-cc-pVTZ basis set with respect to the CCSD(T)/aug-cc-pVTZ energies

All values in kcal mol^-1^

In order to gain additional insight into the quality of the interaction energy curves reproduced by the DFT methods, it is helpful to situate their performances in a ranking covering both the DFT and the MP2-type methods. The comparison of MSE and RMSE in *E*_int_ and *E*_int_^CP^ obtained by the DFT and MP2-type methods indicates that the SCS-MP2 method reproduces the reference interaction energy curves with the greatest accuracy and its performance is practically independent of the choice of basis set. In contrast to the nearly constant performance of SCS-MP2, the errors determined for the DFT methods are in several cases smaller (e.g., MSE(*E*_int_) of ωB97X for SnH_2_-pyridine) but mostly larger than those of SCS-MP2. On this basis it can be concluded that even the best performing density functionals, that is, the RSH ones and those incorporating the dispersion correction, cannot compete with SCS-MP2. On the other hand, they are superior to the conventional MP2 method.

Having evaluated the performance of the DFT methods in reproducing the structure and energetics of the SnH_2_-benzene and SnH_2_-pyridine complexes, we extended the set of investigated complexes in order to test the transferability of the findings made in the previous paragraphs. In consequence, the performance of 14 DFT methods was reassessed using a test set containing five π-complexes of SnX_2_ with benzene (where X = H, F, Cl, Br, I) and five σ-complexes of SnX_2_ with pyridine. We did not manage to obtain the reference structures of SnX_2_-benzene and SnX_2_-pyridine (X = F, Cl, Br, I) at the CCSD level of theory because performing the respective geometry optimizations far exceeded the computational resources available to us. Hence, we employed less computationally demanding methods to produce the reference structures for the whole set of complexes. We obviously made use of the conclusions drawn in the previous section and, therefore, SOS-MP2 was selected for obtaining the reference geometries of the π-complexes whereas SCS-MP2 provided the reference geometries of the σ-complexes. As we remember, for SnH_2_-benzene and SnH_2_-pyridine these two MP2-type methods produced the values of RMSD that were very close to those of CCSD and simultaneously much smaller than those of the DFT methods. Next, the geometries of additional eight complexes of stannylenes containing halogen atoms were optimized using 14 DFT methods. Table 8 summarizes the most important structural and energetic properties of all ten complexes investigated at the ωB97X/aug-cc-pVTZ level of theory. From the results in this table, we see that the complexations of benzene and pyridine by the stannylenes containing halogen atoms are essentially similar to the corresponding complexations by SnH_2_. The structures of the complexes with the stannylenes containing halogen atoms are not far different from the structures of the complexes with SnH_2_ and the differences in the interaction energies between the complexes with X being a halogen atom and the complexes with X = H do not exceed 1.20 kcal mol^-1^. The *d* distance changes monotonously in both types of complexes with X being a halogen atom. Unsurprisingly, this distance elongates when X gets heavier. It is interesting to note that, in contrast to SnH_2_-benzene, the π-complexes with the halogenated stannylenes exhibit a slight shift of the Sn atom toward the center of the benzene ring (*a*_1_ > 90°). The counterpoise correction for the BSSE is small for the DFT methods. In the case of ωB97X/aug-cc-pVTZ this correction weakens the interaction in the σ-complexes by 0.5 kcal mol^-1^ at the most. The effect of the counterpoise correction on the strength of the interaction in the π-complexes is even smaller.

**Table 8:** Selected geometrical (*d*, *a*_1_, and *a*_2_) and energetic (*E*_int_ and *E*_int_^CP^) parameters for five π-complexes of SnX_2_ with benzene and five σ-complexes of SnX_2_ with pyridine calculated at the ωB97X/aug-cc-pVTZ level of theory

X	SnX_2_-benzene					SnX_2_-pyridine				
	*d*	*a*_1_	*a*_2_	*E*_int_	*E*_int_^CP^	*d*	*a*_1_	*a*_2_	*E*_int_	*E*_int_^CP^
H	2.919	88.19	16.11	−9.38	−9.28	2.380	120.24	5.81	−24.07	−23.83
F	2.998	95.89	5.88	−9.81	−9.68	2.366	125.11	14.51	−24.78	−24.28
Cl	3.029	95.81	1.15	−9.67	−9.71	2.374	120.30	4.07	−25.20	−24.85
Br	3.050	96.17	3.96	−9.45	−9.51	2.382	119.32	1.52	−24.95	−24.57
I	3.076	96.23	6.83	−9.00	−9.10	2.387	118.01	1.84	−24.27	−23.93

Distances in Å, angles in °, and energies in kcal mol^-1^

**Fig. 6:**
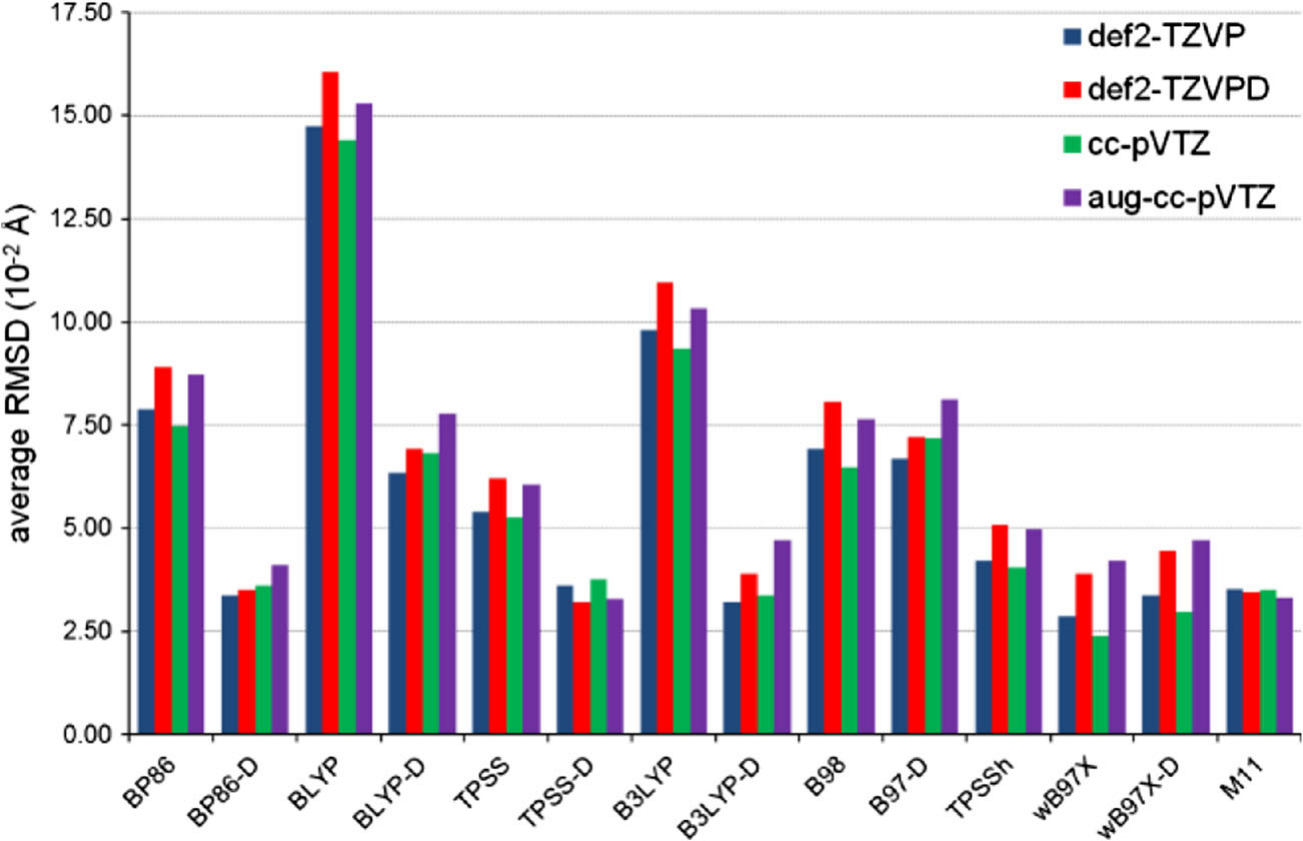
Average RMSD for ten complexes of SnX_2_ (X = H, F, Cl, Br, I) with benzene or pyridine obtained by 14 DFT methods in combination with four basis sets

The structures of the ten complexes optimized by the DFT methods were compared with the corresponding reference structures. For each DFT method combined with a given basis set, the value of RMSD was calculated for each complex and ten resulting RMSD values were averaged in order to express the quality of predicted structures with a single quantity. Figure 6 presents the average values of RMSD for 14 DFT methods and four basis sets. Of the DFT methods without the empirical dispersion correction, ωB97X and M11 are particularly efficient in predicting the structures of the π- and σ-complexes. These density functionals are followed by TPSSh. The inclusion of the dispersion correction to the older density functionals is beneficial, although for BLYP-D its average RMSD value as such still remains large. The comparison of the average RMSD values produced by B98 and B97-D may suggest that the dispersion correction deteriorates the performance of B98. It is not true because in the case of B97-D the effect of the dispersion correction is eliminated by the absence of the HF exchange (while B98 incorporates the HF exchange into its exchange part). The inclusion of the HF exchange leads to a substantial improvement in the agreement with the reference structures, which is indicated by the smaller average RMSD values for B3LYP and TPSSh in juxtaposition with the average RMSD values of BLYP and TPSS, respectively. It is consistent with the findings made previously in this section for the reproduction of the SnH_2_-benzene and SnH_2_-pyridine structures. Overall, the performance of the DFT methods in reproducing the structures of SnH_2_-benzene and SnH_2_-pyridine turns out to be transferable to our more representative set of the complexes of simple stannylenes with aromatic molecules. From the current results and from what we have recently demonstrated for trimethyltin cyanide dimer [91], it can be deduced that the ωB97X and M11 density functionals generally show excellent performance in predicting structures of systems with weak interactions involving tin.

**Fig. 7:**
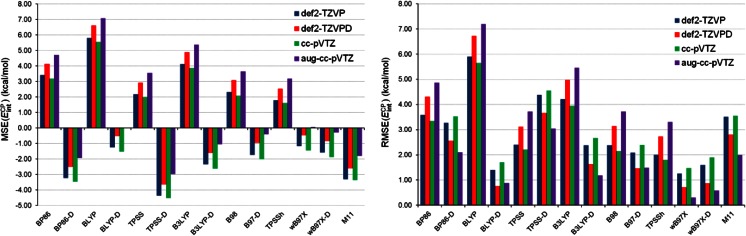
MSE and RMSE in *E*_int_^CP^ obtained by 14 DFT methods in combination with four basis sets with respect to the CCSD(T) results

The next step is to assess the performance of 14 DFT methods using their values of MSE(*E*_int_^CP^) and RMSE(*E*_int_^CP^) with respect to the *E*_int_^CP^ values predicted by CCSD(T). Each DFT method was employed to calculate *E*_int_^CP^ for the structures optimized previously by the same method. The CCSD(T) values of *E*_int_^CP^ were determined for five π-complexes optimized by SOS-MP2 and five σ-complexes optimized by SCS-MP2. Each value of MSE(*E*_int_^CP^) or RMSE(*E*_int_^CP^) was obtained from the calculations utilizing the same basis set. Figure 7 depicts the values of MSE(*E*_int_^CP^) and RMSE(*E*_int_^CP^) for 14 DFT methods in combination with four basis sets. As is seen in the left-hand plot in Fig. 7, non-RSH standard density functionals yield positive MSE(*E*_int_^CP^), which means that the SnX_2_ stannylenes are generally underbound by benzene and pyridine. In contrast to the non-RSH standard density functionals, those utilizing the range separation and/or the dispersion correction tend to overestimate the strength of the interaction between SnX_2_ and benzene/pyridine. Also apparent from the left-hand plot in Fig. 7 is the fact that BLYP-D and ωB97X in conjunction with aug-cc-pVTZ produce negligible MSE(*E*_int_^CP^). These two methods, as well as the ωB97X-D one, afford the best agreements with the CCSD(T) results when RMSE(*E*_int_^CP^) is considered. Admittedly, for these methods their RMSE(*E*_int_^CP^) values are strongly dependent on the basis set employed, but they are always lower than 2.00 kcal mol^-1^. One should, however, remember that the BLYP-D values of *E*_int_^CP^ were calculated for the corresponding optimized geometries that were noticeably different from the reference ones. The opposite situation occurs for ωB97X and ωB97X-D where good geometries correlate with good *E*_int_^CP^ values. The extension of the test set of complexes does not change the main findings made previously for *E*_int_^CP^ in SnH_2_-benzene and SnH_2_-pyridine. In particular, the excellent performance of ωB97X in reproducing *E*_int_^CP^ is confirmed for the set of the SnX_2_-benzene and SnX_2_-pyridine complexes. On the other hand, M11 performs worse for *E*_int_^CP^ in this set compared to its performance for *E*_int_^CP^ in SnH_2_-benzene and SnH_2_-pyridine.

**Fig. 8:**
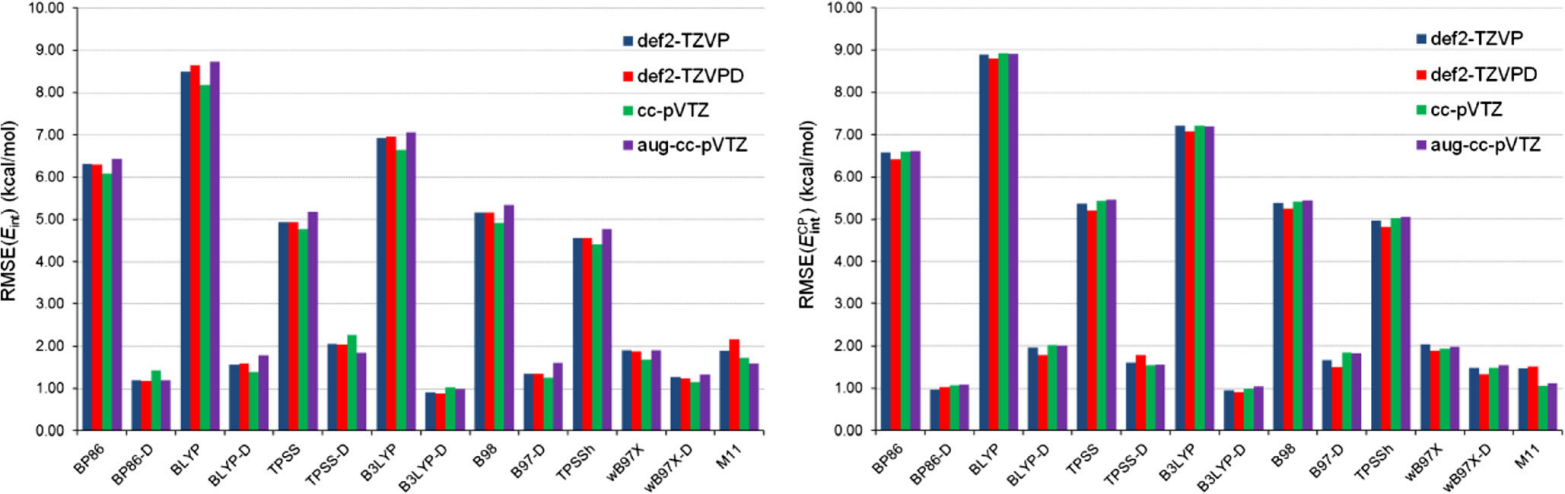
RMSE in *E*_int_ and *E*_int_^CP^ obtained by 14 DFT methods in combination with four basis sets with respect to the SCS-MP2/CBS results

The results presented in Fig. 7 indicate that the performance of individual DFT methods is sensitive to the choice of basis set and an overall assessment of individual DFT methods may sometimes be ambiguous when all four basis sets are taken into consideration. It is mainly due to the fact that our evaluation procedure is based on a comparison to the reference CCSD(T) results obtained with the same triple-ζ basis sets as those combined with the DFT methods. The application of more extended basis sets would undoubtedly be advisable but then the computational cost would become prohibitively expensive. Nevertheless, we have tried to carry out an additional performance assessment of the DFT methods in predicting *E*_int_ and *E*_int_^CP^, and in this study we compare the DFT results to the interaction energies obtained from SCS-MP2 in conjunction with an extrapolation to the complete basis set (CBS) limit (details of the SCS-MP2/CBS calculations are described in section S2, see Electronic supplementary material). The SCS-MP2/CBS determination of the interaction energy in SnX_2_-benzene and SnX_2_-pyridine constituted the highest-quality calculations that were feasible for the computer system available to us. Figure 8 shows RMSE(*E*_int_) and RMSE(*E*_int_^CP^) with respect to the SCS-MP2/CBS results. Immediately apparent are the marked differences in the values of errors between six non-RSH standard density functionals and the other density functionals. The non-RSH standard density functionals yield RMSE(*E*_int_) and RMSE(*E*_int_^CP^) in the range from 4.00 to 9.00 kcal mol^-1^, whereas the RSH and dispersion-corrected density functionals exhibit much lower values of these errors. The performances of the RSH and dispersion-corrected density functionals are actually comparable: their values of errors vary by ca. 1 kcal mol^-1^ at the most. It is also seen from Fig. 8 that there is a considerable reduction of the basis set effect for each DFT method.

## Conclusions

In this work the performances of six MP2-type methods (MP2, SCS-MP2, SOS-MP2, FE2-MP2, SCS(MI)-MP2, and S2-MP2), eight standard density functionals (BP86, BLYP, TPSS, B3LYP, B98, TPSSh, ωB97X, and M11) and their six combinations with an empirical dispersion correction (BP86-D, BLYP-D, TPSS-D, B3LYP-D, B97-D, and ωB97X-D) were assessed for the purposes of investigating the interaction between stannylenes and aromatic molecules. These methods were used in conjunction with four basis sets of triple-ζ valence quality (def2-TZVP(P), def2-TZVP(P)D, cc-pVTZ, and aug-cc-pVTZ). Structural and energetic properties of two kinds of complexes were calculated using the aforementioned methods. The first kind of the studied complexes was formed by a SnX_2_ molecule (where X = H, F, Cl, Br, I) interacting with the π-electrons of benzene. The second one encompassed the σ-complexation of SnX_2_ by the N-atom lone electron pair of pyridine. The assessment of the aforementioned methods was based on several evaluation procedures comparing the structures and interaction energies predicted by these methods with reference computational data. A very detailed analysis of the performances of the MP2-type and DFT methods was carried out for SnH_2_-benzene and SnH_2_-pyridine. The reference structures and interaction energies for these two complexes were obtained from CCSD and CCSD(T), respectively. The DFT methods were also evaluated using the full test set of SnX_2_-benzene and SnX_2_-pyridine complexes but then the reference structures of ten complexes were calculated using the best performing MP2-type methods. Taking into account the results of all evaluation procedures, we make the following findings:

1. Of the MP2-type methods, the reference structure of SnH_2_-benzene is reproduced best by SOS-MP2, whereas SCS-MP2 is capable of mimicking the reference structure of SnH_2_-pyridine with the greatest accuracy. The latter method performs best in predicting the interaction energy between SnH_2_ and benzene and between SnH_2_ and pyridine.
2. Among the DFT methods, ωB97X and M11 provide the structures and interaction energies of the SnH_2_-benzene and SnH_2_-pyridine complexes with good accuracy. Their performances in reproducing the interaction energies are, however, sensitive to the choice of specific triple-ζ basis set.
3. The best performing MP2-type methods are generally better than the DFT methods in reproducing the structures and interaction energies for SnH_2_-benzene and SnH_2_-pyridine. On the other hand, the RSH density functionals and many dispersion-corrected density functionals are often superior to the conventional MP2 method.
4. The evaluation procedure taking five π-complexes and five σ-complexes into account confirms that ωB97X, M11, BP86-D, and TPSS-D are particularly efficient in predicting the structures of these complexes. The good geometries predicted by ωB97X correlate with its good *E*_int_^CP^ values. The comparison of *E*_int_ and *E*_int_^CP^ calculated by 14 DFT methods with the SCS-MP2/CBS results indicates that the levels of accuracy provided by the RSH and the dispersion-corrected density functionals do not differ significantly: the RMSE differences between these density functionals do not exceed 1–2 kcal mol^-1^.

We hope that these findings will provide a guide for the reliable computational investigations of the interaction between stannylenes and aromatic molecules.

## Electronic supplementary material

Below is the link to the electronic supplementary material.

ESM 1 (DOC 366 kb)
