# Supplementary material for: Performance of Møller-Plesset second-order perturbation theory and density functional theory in predicting the interaction between stannylenes and aromatic molecules
Source: J Mol Model. 2015 Feb 13;21(3):41. doi: 10.1007/s00894-015-2589-1 (PMC4326664; doi:10.1007/s00894-015-2589-1)
Supplement: Supplementary file 1 — (DOC 366 kb) [file 894_2015_2589_MOESM1_ESM.doc]

**Electronic supplementary material**

for

Performance of Møller-Plesset second-order perturbation theory and density functional theory in predicting the interaction between stannylenes and aromatic molecules

by

Piotr Matczak^a^ and Sławomir Wojtulewski^b^

^a^ Department of Theoretical and Structural Chemistry, Faculty of Chemistry, University of Łódź, Pomorska 163/165, 90-236 Lodz, Poland

^b^ Institute of Chemistry, University of Białystok, Hurtowa 1, 15-399 Bialystok, Poland

**S1. Calculations of MSE and RMSE**

For the purposes of the analysis of interaction energy curves, the mean signed error (MSE) and the root mean square error (RMSE) in *E*_int_ were calculated using the following formulas:

$\text{MSE}\left(E_{\mathrm{int}}^{\text{MP2}-\text{type}}\right)=\frac{\sum_{i=1}^{n} \left(E_{\mathrm{int},i}^{\text{MP2}-\text{type}}-E_{\mathrm{int},i}^{\text{CCSD(T)}}\right)}{n}$ (S1)

$\text{RMSE}\left(E_{\mathrm{int}}^{\text{MP2}-\text{type}}\right)=\sqrt{\frac{\sum_{i=1}^{n} \left(E_{\mathrm{int},i}^{\text{MP2}-\text{type}}-E_{\mathrm{int},i}^{\text{CCSD(T)}}\right)^{2}}{n}}$ (S2)

where the superscript in *E*_int_ indicates one of the MP2-type methods or the CCSD(T) level of theory and *n* = 13. The same formulas were used for $E_{\mathrm{int}}^{\text{CP}}$. These two statistical quantities were also used for the analysis of the interaction energy curves obtained from fourteen DFT methods and for the assessment of *E*_int_ and $E_{\mathrm{int}}^{\text{CP}}$ calculated by the DFT methods for the test set of 10 complexes (in this case *n* = 10).

**S2. Calculations of interaction energy extrapolated to the CBS limit**

The two-point extrapolation procedure of Halkier et al. [S1,S2] was employed to calculate the total energies of the SnX_2_ (X = H, F, Cl, Br, I), benzene and pyridine molecules as well as of their complexes at the SCS-MP2 level of theory. These total energies were extrapolated to the complete basis set (CBS) limit using the following expression:

$E_{CBS}^{tot}=\frac{e^{\alpha}}{e^{\alpha}-1}E_{4}^{HF}-\frac{1}{e^{\alpha}-1}E_{3}^{HF}+\frac{\frac{64}{27}}{\frac{64}{27}-1}E_{4}^{corr}-\frac{1}{\frac{64}{27}-1}E_{3}^{corr}$ (S3)

where the coefficient α was equal to 1.63. In Eq. (S3) the Hartree-Fock energies *E^HF^* and the correlation energies *E^corr^* were calculated using a pair of triple- and quadruple-ζ basis sets, that is, def2-TZVPP and def2-QZVPP; def2-TZVPPD and def2-QZVPPD; cc-pVTZ and cc-pVQZ; aug-cc-pVTZ and aug-cc-pVQZ. *E*_3_ and *E*_4_ refer to the energies computed with triple- and quadruple-ζ basis sets, respectively. Counterpoise corrections for the BSSE were taken into account in the extrapolated total energies of the individual molecules forming the complexes. The SCS-MP2/CBS interaction energy between SnX_2_ and benzene/pyridine in the complexes was calculated using the $E_{CBS}^{tot}$ energies.

**S3. Additional tables**

**Table S1** Selected geometrical parameters (*d*, *a*_1_ and *a*_2_) for the π-complex of SnH_2_ with benzene and for the σ-complex of SnH_2_ with pyridine optimized by six MP2-type methods and CCSD in combination with the def2-TZVPP basis set. For the MP2-type methods the deviations of the three parameters from the values calculated using CCSD/def2-TZVPP are shown in parentheses

| Method | SnH_2_-benzene | | | SnH_2_-pyridine | | |
| --- | --- | --- | --- | --- | --- | --- |
|  | *d* | *a*_1_ | *a*_2_ | *d* | *a*_1_ | *a*_2_ |
| MP2 | 2.750  (-0.125) | 92.28  (2.98) | 11.40  (-4.23) | 2.325  (-0.026) | 119.90  (-0.39) | 6.08  (0.20) |
| SCS-MP2 | 2.827  (-0.049) | 91.18  (1.88) | 13.10  (-2.53) | 2.349  (-0.002) | 120.17  (-0.12) | 6.07  (0.19) |
| SOS-MP2 | 2.871  (-0.005) | 90.62  (1.32) | 13.95  (-1.69) | 2.362  (0.010) | 120.31  (0.02) | 6.07  (0.20) |
| FE2-MP2 | 3.002  (0.127) | 92.31  (3.01) | 11.53  (-4.11) | 2.328  (-0.023) | 119.99  (-0.31) | 6.24  (0.36) |
| SCS(MI)-MP2 | 2.794  (-0.081) | 90.71  (1.40) | 12.72  (-2.91) | 2.332  (-0.020) | 119.66  (-0.63) | 5.00  (-0.88) |
| S2-MP2 | 2.766  (-0.109) | 92.18  (2.88) | 11.74  (-3.89) | 2.331  (-0.020) | 120.03  (-0.27) | 6.24  (0.36) |
| CCSD | 2.875 | 89.30 | 15.63 | 2.351 | 120.29 | 5.88 |

Distances in Å, angles in °

**Table S2** Selected geometrical parameters (*d*, *a*_1_ and *a*_2_) for the π-complex of SnH_2_ with benzene and for the σ-complex of SnH_2_ with pyridine optimized by six MP2-type methods and CCSD in combination with the def2-TZVPPD basis set. For the MP2-type methods the deviations of the three parameters from the values calculated using CCSD/def2-TZVPPD are shown in parentheses

| Method | SnH_2_-benzene | | | SnH_2_-pyridine | | |
| --- | --- | --- | --- | --- | --- | --- |
|  | *d* | *a*_1_ | *a*_2_ | *d* | *a*_1_ | *a*_2_ |
| MP2 | 2.748  (-0.121) | 93.20  (3.01) | 9.89  (-4.32) | 2.323  (-0.026) | 119.76  (-0.36) | 5.98  (0.30) |
| SCS-MP2 | 2.821  (-0.048) | 92.20  (2.01) | 11.42  (-2.79) | 2.347  (-0.002) | 120.04  (-0.07) | 5.96  (0.28) |
| SOS-MP2 | 2.863  (-0.006) | 91.68  (1.49) | 12.20  (-2.02) | 2.359  (0.010) | 120.18  (0.07) | 5.96  (0.28) |
| FE2-MP2 | 2.755  (-0.114) | 93.27  (3.09) | 9.94  (-4.28) | 2.326  (-0.023) | 119.84  (-0.28) | 6.12  (0.45) |
| SCS(MI)-MP2 | 2.791  (-0.079) | 91.39  (1.20) | 11.62  (-2.60) | 2.330  (-0.018) | 119.59  (-0.53) | 4.97  (-0.71) |
| S2-MP2 | 2.764  (-0.105) | 93.15  (2.97) | 10.13  (-4.09) | 2.329  (-0.020) | 119.88  (-0.24) | 6.13  (0.45) |
| CCSD | 2.869 | 90.19 | 14.21 | 2.349 | 120.11 | 5.68 |

Distances in Å, angles in °

**Table S3** Selected geometrical parameters (*d*, *a*_1_ and *a*_2_) for the π-complex of SnH_2_ with benzene and for the σ-complex of SnH_2_ with pyridine optimized by six MP2-type methods and CCSD in combination with the cc-pVTZ basis set. For the MP2-type methods the deviations of the three parameters from the values calculated using CCSD/cc-pVTZ are shown in parentheses

| Method | SnH_2_-benzene | | | SnH_2_-pyridine | | |
| --- | --- | --- | --- | --- | --- | --- |
|  | *d* | *a*_1_ | *a*_2_ | *d* | *a*_1_ | *a*_2_ |
| MP2 | 2.753  (-0.118) | 91.97  (2.76) | 11.77  (-3.94) | 2.328  (-0.025) | 119.97  (-0.28) | 6.00  (0.09) |
| SCS-MP2 | 2.826  (-0.044) | 90.95  (1.74) | 13.34  (-2.37) | 2.351  (-0.002) | 120.22  (-0.03) | 6.03  (0.12) |
| SOS-MP2 | 2.868  (-0.002) | 90.43  (1.22) | 14.12  (-1.59) | 2.363  (0.010) | 120.35  (0.09) | 6.05  (0.14) |
| FE2-MP2 | 2.759  (-0.111) | 92.00  (2.79) | 11.87  (-3.84) | 2.331  (-0.022) | 120.05  (-0.20) | 6.15  (0.24) |
| SCS(MI)-MP2 | 2.797  (-0.073) | 90.47  (1.26) | 13.08  (-2.62) | 2.336  (-0.017) | 119.73  (-0.52) | 5.02  (-0.88) |
| S2-MP2 | 2.768  (-0.102) | 91.88  (2.67) | 12.07  (-3.64) | 2.334  (-0.019) | 120.09  (-0.16) | 6.16  (0.25) |
| CCSD | 2.870 | 89.21 | 15.71 | 2.353 | 120.25 | 5.91 |

Distances in Å, angles in °

**Table S4** *E*_int_ and $E_{\mathrm{int}}^{\text{CP}}$ for the π-complex of SnH_2_ with benzene and for the σ-complex of SnH_2_ with pyridine obtained by six MP2-type methods and CCSD(T) in combination with the def2-TZVPP basis set. For the MP2-type methods the deviations of their two interaction energies from the values calculated using CCSD(T)/def2-TZVPP are shown in parentheses

| Method | SnH_2_-benzene | | SnH_2_-pyridine | |
| --- | --- | --- | --- | --- |
|  | *E*_int_ | $E_{\mathrm{int}}^{\text{CP}}$ | *E*_int_ | $E_{\mathrm{int}}^{\text{CP}}$ |
| MP2 | -13.29  (-3.22) | -11.08  (-2.84) | -26.80  (-2.34) | -24.41  (-2.20) |
| SCS-MP2 | -10.40  (-0.32) | -8.43  (-0.19) | -24.15  (0.31) | -21.89  (0.32) |
| SOS-MP2 | -9.07  (1.01) | -7.23  (1.01) | -22.86  (1.60) | -20.67  (1.55) |
| FE2-MP2 | -13.05  (-2.97) | -10.75  (-2.51) | -26.52  (-2.06) | -24.05  (-1.83) |
| SCS(MI)-MP2 | -11.22  (-1.15) | -9.87  (-1.62) | -25.32  (-0.87) | -23.75  (-1.53) |
| S2-MP2 | -12.66  (-2.59) | -10.40  (-2.16) | -26.17  (-1.72) | -23.71  (-1.50) |
| CCSD(T) | -10.08 | -8.24 | -24.46 | -22.22 |

All values in kcal/mol

**Table S5** *E*_int_ and $E_{\mathrm{int}}^{\text{CP}}$ for the π-complex of SnH_2_ with benzene and for the σ-complex of SnH_2_ with pyridine obtained by six MP2-type methods and CCSD(T) in combination with the def2-TZVPPD basis set. For the MP2-type methods the deviations of their two interaction energies from the values calculated using CCSD(T)/def2-TZVPPD are shown in parentheses

| Method | SnH_2_-benzene | | SnH_2_-pyridine | |
| --- | --- | --- | --- | --- |
|  | *E*_int_ | $E_{\mathrm{int}}^{\text{CP}}$ | *E*_int_ | $E_{\mathrm{int}}^{\text{CP}}$ |
| MP2 | -14.69  (-3.32) | -11.62  (-2.78) | -27.66  (-2.31) | -24.81  (-2.10) |
| SCS-MP2 | -11.65  (-0.28) | -8.86  (-0.02) | -24.95  (0.40) | -22.23  (0.48) |
| SOS-MP2 | -10.25  (1.12) | -7.60  (1.23) | -23.63  (1.72) | -20.98  (1.73) |
| FE2-MP2 | -14.50  (-3.12) | -11.29  (-2.46) | -27.42  (-2.07) | -24.45  (-1.74) |
| SCS(MI)-MP2 | -12.08  (-0.71) | -10.26  (-1.42) | -25.85  (-0.50) | -24.04  (-1.34) |
| S2-MP2 | -14.09  (-2.72) | -10.92  (-2.09) | -27.07  (-1.72) | -24.11  (-1.40) |
| CCSD(T) | -11.37 | -8.83 | -25.35 | -22.71 |

All values in kcal/mol

**Table S6** *E*_int_ and $E_{\mathrm{int}}^{\text{CP}}$ for the π-complex of SnH_2_ with benzene and for the σ-complex of SnH_2_ with pyridine obtained by six MP2-type methods and CCSD(T) in combination with cc-pVTZ basis set. For the MP2-type methods the deviations of their two interaction energies from the values calculated using CCSD(T)/cc-pVTZ are shown in parentheses

| Method | SnH_2_-benzene | | SnH_2_-pyridine | |
| --- | --- | --- | --- | --- |
|  | *E*_int_ | $E_{\mathrm{int}}^{\text{CP}}$ | *E*_int_ | $E_{\mathrm{int}}^{\text{CP}}$ |
| MP2 | -13.27  (-3.03) | -10.49  (-2.49) | -27.88  (-2.55) | -24.33  (-2.27) |
| SCS-MP2 | -10.42  (-0.18) | -8.05  (-0.05) | -25.14  (0.18) | -21.89  (0.18) |
| SOS-MP2 | -9.11  (1.13) | -6.94  (1.06) | -23.81  (1.51) | -20.69  (1.38) |
| FE2-MP2 | -13.04  (-2.79) | -10.19  (-2.19) | -27.63  (-2.30) | -23.99  (-1.92) |
| SCS(MI)-MP2 | -11.21  (-0.97) | -9.40  (-1.39) | -26.14  (-0.81) | -23.60  (-1.53) |
| S2-MP2 | -12.66  (-2.42) | -9.86  (-1.86) | -27.27  (-1.94) | -23.67  (-1.60) |
| CCSD(T) | -10.24 | -8.00 | -25.33 | -22.07 |

All values in kcal/mol

**Table S7** MSE and RMSE in *E*_int_ and $E_{\mathrm{int}}^{\text{CP}}$ across the interaction energy curves for the π-complex of SnH_2_ with benzene and for the σ-complex of SnH_2_ with pyridine. The errors are calculated for the interaction energies obtained from MP2 and its variants combined with the def2-TZVPP basis set with respect to the CCSD(T)/def2-TZVPP energies

| Method | SnH_2_-benzene | | | | SnH_2_-pyridine | | | |
| --- | --- | --- | --- | --- | --- | --- | --- | --- |
|  | *E*_int_ | | $E_{\mathrm{int}}^{\text{CP}}$ | | *E*_int_ | | $E_{\mathrm{int}}^{\text{CP}}$ | |
|  | MSE | RMSE | MSE | RMSE | MSE | RMSE | MSE | RMSE |
| MP2 | -1.74 | 2.28 | -1.71 | 2.24 | -0.84 | 1.10 | -0.81 | 1.07 |
| SCS-MP2 | -0.20 | 0.22 | -0.19 | 0.22 | 0.11 | 0.20 | 0.11 | 0.20 |
| SOS-MP2 | 0.58 | 0.81 | 0.57 | 0.81 | 0.59 | 0.84 | 0.58 | 0.83 |
| FE2-MP2 | -1.60 | 2.09 | -1.53 | 1.99 | -0.75 | 0.96 | -0.68 | 0.89 |
| SCS(MI)-MP2 | -0.73 | 0.95 | -1.09 | 1.43 | -0.28 | 0.41 | -0.55 | 0.78 |
| S2-MP2 | -1.40 | 1.83 | -1.33 | 1.73 | -0.62 | 0.80 | -0.56 | 0.72 |

All values in kcal/mol

**Table S8** MSE and RMSE in *E*_int_ and $E_{\mathrm{int}}^{\text{CP}}$ across the interaction energy curves for the π-complex of SnH_2_ with benzene and for the σ-complex of SnH_2_ with pyridine. The errors are calculated for the interaction energies obtained from MP2 and its variants combined with the def2-TZVPPD basis set with respect to the CCSD(T)/def2-TZVPPD energies

| Method | SnH_2_-benzene | | | | SnH_2_-pyridine | | | |
| --- | --- | --- | --- | --- | --- | --- | --- | --- |
|  | *E*_int_ | | $E_{\mathrm{int}}^{\text{CP}}$ | | *E*_int_ | | $E_{\mathrm{int}}^{\text{CP}}$ | |
|  | MSE | RMSE | MSE | RMSE | MSE | RMSE | MSE | RMSE |
| MP2 | -1.74 | 2.28 | -1.67 | 2.19 | -0.81 | 1.07 | -0.76 | 1.01 |
| SCS-MP2 | -0.15 | 0.18 | -0.11 | 0.12 | 0.17 | 0.25 | 0.20 | 0.29 |
| SOS-MP2 | 0.64 | 0.88 | 0.68 | 0.93 | 0.66 | 0.91 | 0.68 | 0.94 |
| FE2-MP2 | -1.64 | 2.13 | -1.49 | 1.94 | -0.74 | 0.96 | -0.64 | 0.83 |
| SCS(MI)-MP2 | -0.44 | 0.65 | -0.97 | 1.29 | -0.05 | 0.24 | -0.45 | 0.67 |
| S2-MP2 | -1.44 | 1.86 | -1.29 | 1.67 | -0.61 | 0.79 | -0.51 | 0.66 |

All values in kcal/mol

**Table S9** MSE and RMSE in *E*_int_ and $E_{\mathrm{int}}^{\text{CP}}$ across the interaction energy curves for the π-complex of SnH_2_ with benzene and for the σ-complex of SnH_2_ with pyridine. The errors are calculated for the interaction energies obtained from MP2 and its variants combined with the cc-pVTZ basis set with respect to the CCSD(T)/cc-pVTZ energies

| Method | SnH_2_-benzene | | | | SnH_2_-pyridine | | | |
| --- | --- | --- | --- | --- | --- | --- | --- | --- |
|  | *E*_int_ | | $E_{\mathrm{int}}^{\text{CP}}$ | | *E*_int_ | | $E_{\mathrm{int}}^{\text{CP}}$ | |
|  | MSE | RMSE | MSE | RMSE | MSE | RMSE | MSE | RMSE |
| MP2 | -1.61 | 2.12 | -1.55 | 2.03 | -0.95 | 1.23 | -0.86 | 1.13 |
| SCS-MP2 | -0.10 | 0.11 | -0.11 | 0.12 | 0.05 | 0.13 | 0.05 | 0.13 |
| SOS-MP2 | 0.66 | 0.91 | 0.61 | 0.84 | 0.55 | 0.79 | 0.51 | 0.74 |
| FE2-MP2 | -1.48 | 1.95 | -1.37 | 1.79 | -0.86 | 1.10 | -0.74 | 0.96 |
| SCS(MI)-MP2 | -0.63 | 0.81 | -0.97 | 1.28 | -0.30 | 0.40 | -0.58 | 0.80 |
| S2-MP2 | -1.29 | 1.69 | -1.18 | 1.54 | -0.73 | 0.93 | -0.62 | 0.79 |

All values in kcal/mol

**Table S10** Selected geometrical parameters (*d*, *a*_1_ and *a*_2_) for the π-complex of SnH_2_ with benzene and for the σ-complex of SnH_2_ with pyridine optimized by fourteen DFT methods in combination with the def2-TZVP basis set. The deviations of the three parameters from the values calculated using CCSD/def2-TZVPP are shown in parentheses

| Method | SnH_2_-benzene | | | SnH_2_-pyridine | | |
| --- | --- | --- | --- | --- | --- | --- |
|  | *d* | *a*_1_ | *a*_2_ | *d* | *a*_1_ | *a*_2_ |
| BP86 | 2.841  (-0.034) | 85.37  (-3.93) | 18.54  (2.90) | 2.388  (0.037) | 119.84  (-0.45) | 5.38  (-0.50) |
| BP86-D | 2.837  (-0.038) | 88.34  (-0.96) | 16.31  (0.68) | 2.379  (0.028) | 120.78  (0.49) | 8.00  (2.12) |
| BLYP | 2.992  (0.117) | 81.65  (-7.65) | 22.20  (6.56) | 2.441  (0.090) | 119.97  (-0.32) | 5.09  (-0.79) |
| BLYP-D | 2.952  (0.077) | 86.05  (-3.26) | 18.26  (2.62) | 2.423  (0.072) | 120.85  (0.55) | 7.71  (1.83) |
| TPSS | 2.811  (-0.064) | 86.51  (-2.79) | 18.48  (2.85) | 2.373  (0.022) | 120.30  (0.01) | 6.42  (0.54) |
| TPSS-D | 2.807  (-0.068) | 89.54  (0.24) | 15.95  (0.31) | 2.366  (0.015) | 121.29  (1.00) | 9.08  (3.20) |
| B3LYP | 2.953  (0.078) | 83.59  (-5.71) | 20.45  (4.81) | 2.406  (0.055) | 119.89  (-0.40) | 5.05  (-0.82) |
| B3LYP-D | 2.929  (0.054) | 86.93  (-2.37) | 17.51  (1.87) | 2.396  (0.044) | 120.70  (0.40) | 7.34  (1.46) |
| B98 | 2.905  (0.030) | 85.66  (-3.64) | 18.41  (2.78) | 2.390  (0.039) | 119.90  (-0.39) | 5.19  (-0.68) |
| B97-D | 2.964  (0.089) | 87.41  (-1.89) | 16.11  (0.48) | 2.418  (0.067) | 120.53  (0.24) | 6.93  (1.05) |
| TPSSh | 2.810  (-0.065) | 87.00  (-2.30) | 17.82  (2.18) | 2.364  (0.013) | 120.22  (-0.07) | 6.18  (0.31) |
| ωB97X | 2.923  (0.048) | 88.43  (-0.87) | 15.78  (0.14) | 2.379  (0.028) | 120.21  (-0.09) | 5.58  (-0.30) |
| ωB97X-D | 2.927  (0.052) | 88.46  (-0.84) | 16.31  (0.68) | 2.382  (0.031) | 120.43  (0.14) | 6.20  (0.32) |
| M11 | 2.860  (-0.015) | 90.75  (1.45) | 12.96  (-2.67) | 2.364  (0.012) | 120.65  (0.35) | 6.76  (0.88) |

Distances in Å, angles in °

**Table S11** Selected geometrical parameters (*d*, *a*_1_ and *a*_2_) for the π-complex of SnH_2_ with benzene and for the σ-complex of SnH_2_ with pyridine optimized by fourteen DFT methods in combination with the def2-TZVPD basis set. The deviations of the three parameters from the values calculated using CCSD/def2-TZVPPD are shown in parentheses

| Method | SnH_2_-benzene | | | SnH_2_-pyridine | | |
| --- | --- | --- | --- | --- | --- | --- |
|  | *d* | *a*_1_ | *a*_2_ | *d* | *a*_1_ | *a*_2_ |
| BP86 | 2.839  (-0.030) | 85.19  (-5.00) | 18.76  (4.55) | 2.388  (0.039) | 119.85  (-0.26) | 5.44  (-0.24) |
| BP86-D | 2.835  (-0.034) | 88.25  (-1.93) | 16.45  (2.24) | 2.379  (0.030) | 120.79  (0.68) | 8.06  (2.38) |
| BLYP | 2.991  (0.122) | 81.31  (-8.88) | 22.51  (8.29) | 2.441  (0.092) | 119.97  (-0.15) | 5.14  (-0.54) |
| BLYP-D | 2.950  (0.081) | 85.86  (-4.32) | 18.45  (4.23) | 2.423  (0.074) | 120.85  (0.74) | 7.77  (2.09) |
| TPSS | 2.810  (-0.059) | 86.38  (-3.80) | 18.66  (4.44) | 2.373  (0.024) | 120.32  (0.21) | 6.51  (0.84) |
| TPSS-D | 2.805  (-0.064) | 89.49  (-0.70) | 16.05  (1.84) | 2.366  (0.017) | 121.32  (1.20) | 9.19  (3.51) |
| B3LYP | 2.951  (0.082) | 83.30  (-6.88) | 20.72  (6.50) | 2.406  (0.057) | 119.90  (-0.22) | 5.10  (-0.58) |
| B3LYP-D | 2.927  (0.058) | 86.75  (-3.44) | 17.69  (3.47) | 2.395  (0.047) | 120.70  (0.59) | 7.39  (1.71) |
| B98 | 2.903  (0.034) | 85.45  (-4.74) | 18.63  (4.42) | 2.390  (0.042) | 119.91  (-0.20) | 5.25  (-0.43) |
| B97-D | 2.963  (0.093) | 87.25  (-2.94) | 16.32  (2.10) | 2.418  (0.069) | 120.54  (0.42) | 6.99  (1.31) |
| TPSSh | 2.808  (-0.061) | 86.88  (-3.31) | 17.98  (3.76) | 2.364  (0.015) | 120.24  (0.12) | 6.27  (0.60) |
| ωB97X | 2.920  (0.051) | 88.19  (-1.99) | 15.93  (1.72) | 2.377  (0.028) | 120.18  (0.07) | 5.56  (-0.12) |
| ωB97X-D | 2.924  (0.055) | 88.30  (-1.88) | 16.45  (2.24) | 2.381  (0.033) | 120.44  (0.33) | 6.24  (0.56) |
| M11 | 2.864  (-0.006) | 91.03  (0.85) | 12.60  (-1.61) | 2.364  (0.015) | 120.73  (0.61) | 6.95  (1.27) |

Distances in Å, angles in °

**Table S12** Selected geometrical parameters (*d*, *a*_1_ and *a*_2_) for the π-complex of SnH_2_ with benzene and for the σ-complex of SnH_2_ with pyridine optimized by fourteen DFT methods in combination with the cc-pVTZ basis set. The deviations of the three parameters from the values calculated using CCSD/cc-pVTZ are shown in parentheses

| Method | SnH_2_-benzene | | | SnH_2_-pyridine | | |
| --- | --- | --- | --- | --- | --- | --- |
|  | *d* | *a*_1_ | *a*_2_ | *d* | *a*_1_ | *a*_2_ |
| BP86 | 2.842  (-0.029) | 85.09  (-4.12) | 19.02  (3.32) | 2.394  (0.041) | 119.91  (-0.34) | 5.69  (-0.22) |
| BP86-D | 2.837  (-0.033) | 88.16  (-1.05) | 16.71  (1.00) | 2.384  (0.031) | 120.86  (0.61) | 8.32  (2.42) |
| BLYP | 2.991  (0.121) | 81.37  (-7.84) | 22.65  (6.94) | 2.447  (0.094) | 120.06  (-0.20) | 5.42  (-0.49) |
| BLYP-D | 2.952  (0.081) | 85.93  (-3.28) | 18.57  (2.86) | 2.428  (0.075) | 120.94  (0.69) | 8.06  (2.16) |
| TPSS | 2.811  (-0.059) | 86.13  (-3.08) | 19.12  (3.41) | 2.379  (0.025) | 120.37  (0.12) | 6.74  (0.84) |
| TPSS-D | 2.807  (-0.063) | 89.22  (0.01) | 16.53  (0.82) | 2.371  (0.018) | 121.38  (1.12) | 9.42  (3.51) |
| B3LYP | 2.952  (0.082) | 83.42  (-5.78) | 20.77  (5.06) | 2.412  (0.059) | 119.96  (-0.29) | 5.37  (-0.54) |
| B3LYP-D | 2.929  (0.058) | 86.86  (-2.35) | 17.74  (2.03) | 2.401  (0.048) | 120.78  (0.52) | 7.66  (1.76) |
| B98 | 2.906  (0.035) | 85.48  (-3.73) | 18.78  (3.07) | 2.397  (0.044) | 119.98  (-0.27) | 5.55  (-0.36) |
| B97-D | 2.963  (0.092) | 87.34  (-1.86) | 16.37  (0.66) | 2.423  (0.070) | 120.60  (0.35) | 7.23  (1.33) |
| TPSSh | 2.810  (-0.061) | 86.68  (-2.53) | 18.38  (2.67) | 2.370  (0.017) | 120.28  (0.03) | 6.50  (0.59) |
| ωB97X | 2.920  (0.050) | 88.42  (-0.78) | 15.87  (0.16) | 2.383  (0.029) | 120.22  (-0.03) | 5.80  (-0.10) |
| ωB97X-D | 2.926  (0.056) | 88.49  (-0.72) | 16.37  (0.66) | 2.387  (0.034) | 120.48  (0.22) | 6.45  (0.54) |
| M11 | 2.854  (-0.017) | 90.53  (1.32) | 13.37  (-2.34) | 2.369  (0.016) | 120.62  (0.37) | 6.84  (0.94) |

Distances in Å, angles in °

**Table S13** *E*_int_ and $E_{\mathrm{int}}^{\text{CP}}$ for the π-complex of SnH_2_ with benzene and for the σ-complex of SnH_2_ with pyridine optimized by fourteen DFT methods in combination with the def2-TZVP basis set. The deviations of the two interaction energies from the values calculated using CCSD(T)/def2-TZVPP are shown in parentheses

| Method | SnH_2_-benzene | | SnH_2_-pyridine | |
| --- | --- | --- | --- | --- |
|  | *E*_int_ | $E_{\mathrm{int}}^{\text{CP}}$ | *E*_int_ | $E_{\mathrm{int}}^{\text{CP}}$ |
| BP86 | -6.64  (3.44) | -6.60  (1.64) | -21.50  (2.95) | -21.17  (1.05) |
| BP86-D | -12.52  (-2.44) | -12.29  (-4.05) | -25.88  (-1.42) | -25.54  (-3.33) |
| BLYP | -4.18  (5.90) | -4.10  (4.14) | -18.65  (5.81) | -18.24  (3.97) |
| BLYP-D | -10.51  (-0.43) | -10.26  (-2.02) | -23.52  (0.94) | -23.11  (-0.90) |
| TPSS | -7.51  (2.57) | -7.30  (0.94) | -22.66  (1.80) | -22.30  (-0.09) |
| TPSS-D | -13.08  (-3.00) | -12.86  (-4.62) | -26.89  (-2.43) | -26.53  (-4.31) |
| B3LYP | -4.98  (5.10) | -4.92  (3.32) | -19.80  (4.66) | -19.47  (2.74) |
| B3LYP-D | -10.69  (-0.61) | -10.48  (-2.24) | -24.14  (0.32) | -23.80  (-1.59) |
| B98 | -6.79  (3.29) | -6.75  (1.49) | -20.97  (3.49) | -20.86  (1.36) |
| B97-D | -11.00  (-0.92) | -10.89  (-2.65) | -23.29  (1.16) | -22.94  (-0.72) |
| TPSSh | -7.51  (2.57) | -7.32  (0.92) | -22.67  (1.79) | -22.34  (-0.12) |
| ωB97X | -9.30  (0.78) | -9.21  (-0.97) | -24.08  (0.38) | -23.78  (-1.57) |
| ωB97X-D | -9.88  (0.20) | -9.80  (-1.56) | -23.84  (0.62) | -23.54  (-1.32) |
| M11 | -10.22  (-0.14) | -9.80  (-1.56) | -24.27  (0.19) | -23.72  (-1.50) |

All values in kcal/mol

**Table S14** *E*_int_ and $E_{\mathrm{int}}^{\text{CP}}$ for the π-complex of SnH_2_ with benzene and for the σ-complex of SnH_2_ with pyridine optimized by fourteen DFT methods in combination with the def2-TZVPD basis set. The deviations of the two interaction energies from the values calculated using CCSD(T)/def2-TZVPPD are shown in parentheses

| Method | SnH_2_-benzene | | SnH_2_-pyridine | |
| --- | --- | --- | --- | --- |
|  | *E*_int_ | $E_{\mathrm{int}}^{\text{CP}}$ | *E*_int_ | $E_{\mathrm{int}}^{\text{CP}}$ |
| BP86 | -6.57  (4.80) | -6.58  (2.25) | -21.33  (4.02) | -21.18  (1.53) |
| BP86-D | -12.44  (-1.07) | -12.27  (-3.43) | -25.71  (-0.36) | -25.55  (-2.84) |
| BLYP | -4.04  (7.33) | -4.04  (4.80) | -18.36  (6.99) | -18.21  (4.50) |
| BLYP-D | -10.36  (1.01) | -10.19  (-1.36) | -23.24  (2.11) | -23.08  (-0.37) |
| TPSS | -7.43  (3.94) | -7.28  (1.56) | -22.45  (2.91) | -22.30  (0.41) |
| TPSS-D | -13.00  (-1.63) | -12.84  (-4.01) | -26.67  (-1.32) | -26.53  (-3.82) |
| B3LYP | -4.87  (6.50) | -4.87  (3.96) | -19.59  (5.77) | -19.45  (3.25) |
| B3LYP-D | -10.58  (0.80) | -10.43  (-1.60) | -23.92  (1.43) | -23.79  (-1.08) |
| B98 | -6.68  (4.69) | -6.70  (2.13) | -21.16  (4.19) | -20.87  (1.84) |
| B97-D | -10.86  (0.51) | -10.81  (-1.98) | -23.04  (2.31) | -22.91  (-0.20) |
| TPSSh | -7.45  (3.93) | -7.30  (1.53) | -22.48  (2.87) | -22.34  (0.37) |
| ωB97X | -9.29  (2.08) | -9.23  (-0.39) | -23.99  (1.36) | -23.84  (-1.13) |
| ωB97X-D | -9.84  (1.53) | -9.80  (-0.96) | -23.70  (1.65) | -23.56  (-0.85) |
| M11 | -10.54  (0.83) | -9.93  (-1.10) | -24.13  (1.22) | -23.67  (-0.96) |

All values in kcal/mol

**Table S15** *E*_int_ and $E_{\mathrm{int}}^{\text{CP}}$ for the π-complex of SnH_2_ with benzene and for the σ-complex of SnH_2_ with pyridine optimized by fourteen DFT methods in combination with the cc-pVTZ basis set. The deviations of the two interaction energies from the values calculated using CCSD(T)/cc-pVTZ are shown in parentheses

| Method | SnH_2_-benzene | | SnH_2_-pyridine | |
| --- | --- | --- | --- | --- |
|  | *E*_int_ | $E_{\mathrm{int}}^{\text{CP}}$ | *E*_int_ | $E_{\mathrm{int}}^{\text{CP}}$ |
| BP86 | -6.63  (3.61) | -6.58  (1.42) | -21.81  (3.52) | -21.05  (1.02) |
| BP86-D | -12.51  (-2.27) | -12.28  (-4.27) | -26.19  (-0.87) | -25.43  (-3.36) |
| BLYP | -4.27  (5.97) | -4.13  (3.87) | -19.08  (6.25) | -18.15  (3.92) |
| BLYP-D | -10.60  (-0.36) | -10.29  (-2.29) | -23.96  (1.37) | -23.02  (-0.95) |
| TPSS | -7.43  (2.81) | -7.20  (0.81) | -22.94  (2.39) | -22.15  (-0.08) |
| TPSS-D | -12.99  (-2.75) | -12.75  (-4.74) | -27.17  (-1.84) | -26.38  (-4.31) |
| B3LYP | -5.04  (5.20) | -4.93  (3.08) | -20.14  (5.19) | -19.36  (2.71) |
| B3LYP-D | -10.75  (-0.51) | -10.50  (-2.49) | -24.48  (0.85) | -23.69  (-1.62) |
| B98 | -6.79  (3.45) | -6.71  (1.29) | -21.44  (3.88) | -20.74  (1.33) |
| B97-D | -11.05  (-0.80) | -10.88  (-2.88) | -23.65  (1.67) | -22.83  (-0.77) |
| TPSSh | -7.43  (2.81) | -7.21  (0.79) | -22.92  (2.41) | -22.19  (-0.12) |
| ωB97X | -9.39  (0.85) | -9.26  (-1.26) | -24.43  (0.90) | -23.74  (-1.67) |
| ωB97X-D | -9.93  (0.31) | -9.83  (-1.83) | -24.15  (1.18) | -23.47  (-1.40) |
| M11 | -9.92  (0.32) | -9.59  (-1.59) | -24.15  (1.17) | -23.35  (-1.29) |

All values in kcal/mol

**Table S16** MSE and RMSE in *E*_int_ and $E_{\mathrm{int}}^{\text{CP}}$ across the interaction energy curves for the π-complex of SnH_2_ with benzene and for the σ-complex of SnH_2_ with pyridine. The errors are calculated for the interaction energies obtained from fourteen DFT methods combined with the def2-TZVP basis set with respect to the CCSD(T)/def2-TZVPP energies

| Method | SnH_2_-benzene | | | | SnH_2_-pyridine | | | |
| --- | --- | --- | --- | --- | --- | --- | --- | --- |
|  | *E*_int_ | | $E_{\mathrm{int}}^{\text{CP}}$ | | *E*_int_ | | $E_{\mathrm{int}}^{\text{CP}}$ | |
|  | MSE | RMSE | MSE | RMSE | MSE | RMSE | MSE | RMSE |
| BP86 | 2.60 | 2.80 | 1.62 | 1.65 | 1.40 | 1.66 | 0.68 | 0.74 |
| BP86-D | -1.01 | 1.39 | -1.98 | 2.59 | -0.81 | 1.05 | -1.52 | 1.91 |
| BLYP | 4.22 | 5.15 | 3.27 | 3.89 | 2.22 | 3.10 | 1.56 | 2.17 |
| BLYP-D | 0.10 | 1.41 | -0.85 | 1.30 | -0.31 | 1.16 | -0.96 | 1.25 |
| TPSS | 2.00 | 2.17 | 1.02 | 1.11 | 0.88 | 1.07 | 0.17 | 0.21 |
| TPSS-D | -1.43 | 1.74 | -2.41 | 3.01 | -1.23 | 1.41 | -1.93 | 2.32 |
| B3LYP | 3.45 | 4.16 | 2.46 | 2.87 | 1.92 | 2.47 | 1.20 | 1.49 |
| B3LYP-D | -0.16 | 1.04 | -1.14 | 1.41 | -0.29 | 0.69 | -1.01 | 1.17 |
| B98 | 2.08 | 2.58 | 1.08 | 1.27 | 1.20 | 1.66 | 0.45 | 0.66 |
| B97-D | -0.56 | 1.44 | -1.54 | 1.83 | -0.35 | 1.17 | -1.05 | 1.26 |
| TPSSh | 1.95 | 2.10 | 0.95 | 1.07 | 0.99 | 1.13 | 0.26 | 0.31 |
| ωB97X | 0.48 | 1.04 | -0.53 | 0.68 | -0.17 | 0.46 | -0.92 | 1.13 |
| ωB97X-D | -0.08 | 0.99 | -1.08 | 1.18 | -0.20 | 0.54 | -0.95 | 1.05 |
| M11 | 0.42 | 0.48 | -0.45 | 0.94 | 0.24 | 0.31 | -0.41 | 0.75 |

All values in kcal/mol

**Table S17** MSE and RMSE in *E*_int_ and $E_{\mathrm{int}}^{\text{CP}}$ across the interaction energy curves for the π-complex of SnH_2_ with benzene and for the σ-complex of SnH_2_ with pyridine. The errors are calculated for the interaction energies obtained from fourteen DFT methods combined with the def2-TZVPD basis set with respect to the CCSD(T)/def2-TZVPPD energies

| Method | SnH_2_-benzene | | | | SnH_2_-pyridine | | | |
| --- | --- | --- | --- | --- | --- | --- | --- | --- |
|  | *E*_int_ | | $E_{\mathrm{int}}^{\text{CP}}$ | | *E*_int_ | | $E_{\mathrm{int}}^{\text{CP}}$ | |
|  | MSE | RMSE | MSE | RMSE | MSE | RMSE | MSE | RMSE |
| BP86 | 3.47 | 3.77 | 2.01 | 2.07 | 2.07 | 2.37 | 0.96 | 1.03 |
| BP86-D | -0.13 | 0.60 | -1.59 | 2.16 | -0.13 | 0.50 | -1.24 | 1.61 |
| BLYP | 5.13 | 6.14 | 3.67 | 4.34 | 2.97 | 3.84 | 1.86 | 2.48 |
| BLYP-D | 1.01 | 1.94 | -0.44 | 1.06 | 0.45 | 1.33 | -0.66 | 1.01 |
| TPSS | 2.88 | 3.15 | 1.41 | 1.50 | 1.57 | 1.81 | 0.45 | 0.47 |
| TPSS-D | -0.55 | 0.79 | -2.02 | 2.57 | -0.53 | 0.68 | -1.65 | 2.00 |
| B3LYP | 4.34 | 5.14 | 2.86 | 3.32 | 2.61 | 3.19 | 1.49 | 1.80 |
| B3LYP-D | 0.74 | 1.44 | -0.74 | 1.03 | 0.41 | 0.85 | -0.72 | 0.86 |
| B98 | 2.97 | 3.56 | 1.48 | 1.73 | 1.86 | 2.34 | 0.73 | 0.95 |
| B97-D | 0.35 | 1.60 | -1.13 | 1.46 | 0.37 | 1.34 | -0.76 | 1.03 |
| TPSSh | 2.82 | 3.07 | 1.34 | 1.44 | 1.66 | 1.86 | 0.54 | 0.56 |
| ωB97X | 1.30 | 1.80 | -0.18 | 0.44 | 0.44 | 0.66 | -0.68 | 0.86 |
| ωB97X-D | 0.76 | 1.52 | -0.71 | 0.85 | 0.44 | 0.80 | -0.68 | 0.76 |
| M11 | 1.08 | 1.14 | -0.13 | 0.66 | 0.90 | 0.95 | -0.06 | 0.51 |

All values in kcal/mol

**Table S18** MSE and RMSE in *E*_int_ and $E_{\mathrm{int}}^{\text{CP}}$ across the interaction energy curves for the π-complex of SnH_2_ with benzene and for the σ-complex of SnH_2_ with pyridine. The errors are calculated for the interaction energies obtained from fourteen DFT methods combined with the cc-pVTZ basis set with respect to the CCSD(T)/cc-pVTZ energies

| Method | SnH_2_-benzene | | | | SnH_2_-pyridine | | | |
| --- | --- | --- | --- | --- | --- | --- | --- | --- |
|  | *E*_int_ | | $E_{\mathrm{int}}^{\text{CP}}$ | | *E*_int_ | | $E_{\mathrm{int}}^{\text{CP}}$ | |
|  | MSE | RMSE | MSE | RMSE | MSE | RMSE | MSE | RMSE |
| BP86 | 2.65 | 2.88 | 1.45 | 1.48 | 1.57 | 1.93 | 0.69 | 0.73 |
| BP86-D | -0.95 | 1.30 | -2.15 | 2.81 | -0.63 | 0.86 | -1.51 | 1.89 |
| BLYP | 4.22 | 5.19 | 3.08 | 3.65 | 2.32 | 3.32 | 1.57 | 2.18 |
| BLYP-D | 0.10 | 1.47 | -1.03 | 1.40 | -0.20 | 1.30 | -0.95 | 1.21 |
| TPSS | 2.09 | 2.30 | 0.90 | 1.05 | 1.06 | 1.36 | 0.19 | 0.21 |
| TPSS-D | -1.34 | 1.60 | -2.53 | 3.17 | -1.04 | 1.17 | -1.91 | 2.29 |
| B3LYP | 3.46 | 4.21 | 2.29 | 2.64 | 2.08 | 2.73 | 1.21 | 1.49 |
| B3LYP-D | -0.14 | 1.08 | -1.31 | 1.57 | -0.12 | 0.79 | -0.99 | 1.14 |
| B98 | 2.12 | 2.67 | 0.93 | 1.07 | 1.39 | 1.96 | 0.46 | 0.66 |
| B97-D | -0.53 | 1.48 | -1.70 | 1.95 | -0.19 | 1.31 | -1.03 | 1.23 |
| TPSSh | 2.03 | 2.22 | 0.83 | 1.02 | 1.19 | 1.42 | 0.27 | 0.33 |
| ωB97X | 0.47 | 1.06 | -0.73 | 0.87 | 0.00 | 0.53 | -0.94 | 1.16 |
| ωB97X-D | -0.07 | 1.04 | -1.28 | 1.37 | -0.01 | 0.66 | -0.96 | 1.07 |
| M11 | 0.59 | 0.63 | -0.56 | 1.10 | 0.66 | 0.73 | -0.27 | 0.64 |

All values in kcal/mol

**S4. References**

S1. Halkier A, Helgaker T, Jørgensen P, Klopper W, Koch H, Olsen J, Wilson AK (1998) Basis-set convergence in correlated calculations on Ne, N_2_, and H_2_O. Chem Phys Lett 286:243-252

S2. Halkier A, Helgaker T, Jørgensen P, Klopper W, Olsen J (1999) Basis-set convergence of the energy in molecular Hartree-Fock calculations. Chem Phys Lett 302:437-446
